# Cerebrospinal Fluid Biomarkers Are Associated With Glial Fibrillary Acidic Protein and αII-spectrin Breakdown Products in Brain Tissues Following Penetrating Ballistic-Like Brain Injury in Rats

**DOI:** 10.3389/fneur.2018.00490

**Published:** 2018-07-04

**Authors:** Kristen E. DeDominicis, Hye Hwang, Casandra M. Cartagena, Deborah A. Shear, Angela M. Boutté

**Affiliations:** Brain Trauma Neuroprotection and Neurorestoration Branch, Center for Military Psychiatry and Neuroscience, Walter Reed Army Institute of Research, Silver Spring, MD, United States

**Keywords:** traumatic brain injury, biomarker, glial fibrillary acidic protein, αII-spectrin, breakdown product, penetrating ballistic-like brain injury, subacute, chronic

## Abstract

Treatments to improve outcomes following severe traumatic brain injury (TBI) are limited but may benefit from understanding subacute-chronic brain protein profiles and identifying biomarkers suitable for use in this time. Acute alterations in the well-known TBI biomarkers glial fibrillary acidic protein (GFAP), αII-spectrin, and their breakdown products (BDPs) have been well established, but little is known about the subacute-chronic post-injury profiles of these biomarkers. Thus, the current study was designed to determine the extended profile of these TBI-specific biomarkers both in brain tissue and cerebral spinal fluid (CSF). Protein abundance was evaluated in brain tissue samples taken from regions of interest and in CSF at 24 h, 3 days, 7 days, 1 month, and 3 months following severe TBI in rats. Results showed increased full length GFAP (GFAP-FL) and GFAP-BDPs starting at 24 h that remained significantly elevated in most brain regions out to 3 months post-injury. However, in CSF, neither GFAP-FL nor GFAP-BDPs were elevated as a consequence of injury. Regional-specific reduction in αII-spectrin was evident in brain tissue samples from 24 h through 3 months. In contrast, SBDP-145/150 was robustly elevated in most brain regions and in CSF from 24 h through 7 days. Correlation analyses revealed numerous significant relationships between proteins in CSF and brain tissue or neurological deficits. This work indicates that TBI results in chronic changes in brain protein levels of well-known TBI biomarkers GFAP, αII-spectrin, and their BDPs and that SBDP-145/150 may have utility as an acute-chronic biomarker.

## Introduction

Severe penetrating traumatic brain injury (TBI) from gunshot wounds is of concern to both military and civilians alike. Approximately 5,000 penetrating TBI cases were reported from 2000 to 2017 among military personnel (1). Additionally, a high incidence of gun violence persists among civilian populations (2). Severe TBI patients are at risk for mortality and reduced life expectancy (3–5) and the incidence and prevalence of chronic debilitating disability among severe TBI patients are substantial (6–8). Further, the lifetime costs of long-term treatment and care for a severely injured TBI patient and the stresses placed on family caretakers are devastating. Neurosurgical interventions may provide benefits such as reduced intracranial pressure (9) and decreased mortality (10). Yet, clinically effective treatments for treating severe TBI at the molecular level remain somewhat limited, which constitutes a critical gap in patient care. Increased understanding of acute-chronic molecular events in multiple brain regions following severe TBI is important to provide guidance regarding targeted solutions for both acute and chronic treatments.

Traumatic brain injury results in a primary injury characterized by immediate destruction of brain tissue with hemorrhage followed by a complex secondary injury cascade during which there is an influx of intracellular calcium (11–13) and subsequent activation of calcium-activated, non-lysosomal proteases such as calpain-II (14). Glial fibrillary acidic protein (GFAP), an intermediate filament protein expressed abundantly by astrocytes that is increased in multiple TBI models during reactive astrogliosis (15, 16), is a well described substrate of calpain-II mediated proteolysis. Another key substrate, non-erythroid αII-spectrin, is a neuronal scaffolding protein expressed abundantly in axons and pre-synaptic terminals (17). Cleavage of GFAP and αII-spectrin by calpain-II results in generation of breakdown products (BDPs) (18, 19). These proteins are highly enriched throughout the CNS and are detectable in biofluids following TBI. Therefore, these proteins and their BDPs have garnered significant interest for their potential utility as indicators of TBI-mediated astrogliosis and neuronal cell death during secondary brain injury cascades and as peripheral biomarkers associated with focal injury severity and negative outcomes (20–22). It should be noted, however, that astrogliosis is not necessarily a maladaptive process and may provide benefit following TBI (23).

The increased presence of these proteins and their BDPs in brain tissues has been well described following acute TBI (24–27). However, these observations may persist chronically and differ across brain regions and CSF. GFAP-BDPs (28) and GFAP autoantibodies (29) are increased in blood derived from chronic TBI patients. CSF levels of αII-spectrin breakdown products (SBDPs) remain elevated compared to controls at 5–7 days after severe penetrating TBI (20), suggesting this increase may continue past the acute timeframe. Additionally, the relative abundance of calpain-II is rarely described within matching cohorts over an extended period although it is a key mediator of BDP generation. Longitudinal characterization of these proteins in brain tissues would provide vital information regarding the duration and extent of secondary injury following severe TBI, while temporal alteration of these proteins in biofluids may prove useful as biomarkers to track injury progression. Further, defining how this secondary injury cascade differs across brain regions that are either proximal or distal to the injury trajectory site as a consequence of both time and injury is of critical importance.

The goal of the current study was to temporally define the abundance of GFAP, αII-spectrin, and their associated BDPs in specific brain tissue regions and CSF following TBI. Injuries were induced using a rat model of penetrating ballistic-like brain injury (PBBI), which mimics the permanent injury tract and temporary cavity generated from a penetrating ballistic round (30–32) and results in well-described pathophysiology as well as behavioral impairment (33–35). This study also explores protein levels of calpain-II in brain tissues. We report that calpain-II mediated proteolysis in brain tissues persists well past the acute post injury window and into the chronic time frame following PBBI. Furthermore, we present evidence that detection of αII-spectrin BDPs is prominent in cerebral spinal fluid throughout acute-subacute injury and has value as a correlative biomarker for these same fragments in brain tissues.

## Materials and methods

### Animals

Adult male Sprague-Dawley rats (Charles River Laboratories, Raleigh, VA, United States) weighing ~250–300 g were used in this study. All rats were singly housed with a 12 h normal light/dark cycle. Surgical procedures were performed under isoflurane anesthesia (2–5% delivered in oxygen). For terminal bio-sample collections at 24 hours (h), 3 days (d), 7 days, 1 month (m), and 3m following injury, rats were deeply anesthetized with 70 mg/kg ketamine and 6 mg/kg xylazine. Research was conducted under an approved animal use protocol in an AAALACi accredited facility in compliance with the Animal Welfare Act and other federal statutes and regulations relating to animals and experiments involving animals and adheres to principles stated in the *Guide for the Care and Use of Laboratory Animals*, NRC Publication, 2011 edition.

### Brain injury procedures

The WRAIR PBBI model captures the injury trajectory and temporary cavity generated by energy dissipation from a high-energy bullet wound to the head (30). Unilateral frontal PBBI was induced as previously described (34). Briefly, anesthetized rats were placed on a stereotaxic frame. Following craniotomy (+4.5 mm antero-posterior, +2 mm medio-lateral from bregma), a probe was inserted through the right frontal cortex and striatum to 1.2 cm from dura and a computer-controlled pulse generator was activated to rapidly inflate and deflate a balloon on the end of the probe, creating a temporary cavity equivalent to 10% brain volume. Probe injured rats received identical procedures except balloon inflation/deflation. Sham rats received craniotomy without probe insertion.

### Neuroscore assessment

Neurological deficits were assessed using a modified testing paradigm (36) at 24 h following injury or sham manipulation by an experimenter who was blinded to the injury condition. A composite score was generated from four separate parameters: contralateral forelimb flexion, body upward curling behavior during tail suspension, open-field circling behavior, and impaired resistance to lateral push. Each parameter was scored from 0 (normal) to 3 (severely impaired) for a maximum composite score of 12 for each animal.

### Cerebral spinal fluid and brain tissue collection and preparation

A 4-cm midline incision was made from 0.5 cm anterior to the interauricular line. The atlanto-occipital dura mater was exposed by separating the nuchal muscles and CSF was collected with a 30-gauge syringe needle through the membrane. CSF was stored on ice, supplemented with 1xHALT protease/phosphatase inhibitor mix (Thermo Fisher Scientific, Grand Island, NY, United States), and centrifuged at 1,400 *g* for 10 min at 4°C. The resulting cell free CSF was stored at −80°C until use. To increase the concentration of antigens in CSF, the entire collected volume of each CSF sample was concentrated with Amicon Ultra Centrifugal Filter Units with Ultracel-3 membranes (EMD Millipore, Billerica, MA, United States) for 30 min at 14,000 × *g* at 4°C (normalization method presented in “Statistical Analysis”). Ipsilateral brain tissues were washed with 0.9% saline and dissected on ice to isolate the frontal cortex, striatum, hippocampus, and residual midbrain. All tissues were flash-frozen in liquid nitrogen. Concentrated CSF and flash-frozen brain tissues were stored at −80°C until use.

### Western blotting

Brain tissues were sonicated in 1xRIPA buffer containing 1xHALT protease/phosphatase inhibitors (Thermo Fisher Scientific, Grand Island, NY, United States). Protein concentrations were determined using the BCA assay kit (Thermo Fisher Scientific, Grand Island, NY, United States) and crude homogenates were stored frozen until use. All samples for western blotting were prepared in 1x LDS NuPAGE sample buffer and 250 μM DTT and denatured for 5 min at 95°C. Equivalent amounts of total protein (20 μg for calpain-II, 25 μg for GFAP and αII-spectrin) for tissue samples or 10 μL of concentrated CSF prepared as described above were loaded onto 12% (GFAP in tissue) or 4–12% bis-tris gels (GFAP in CSF, αII-spectrin in tissues and CSF, calpain-II in tissues) for separation by SDS-PAGE (Invitrogen NuPAGE, Carlsbad, CA, United States). Proteins were transferred to PVDF membranes and blocked in 5% milk in 1xPBS (GFAP and αII-spectrin in tissues) or to nitrocellulose membranes and blocked with Odyssey Blocking Buffer – PBS (GFAP and αII-spectrin in CSF, calpain-II in tissues; LI-COR Biosciences, Inc., Lincoln, NE, United States). Individual membranes were probed with the following primary antibodies: GFAP (ab7260, Abcam, Cambridge, MA, United States), αII-spectrin (in tissues: MAB1622, EMD Millipore, Billerica, MA, United States; in CSF: BML-FG6090, ENZO, Farmingdale, NY, United States), Calpain-II – Large Subunit (#2539, Cell Signaling Technology, Danvers, MA, United States), and washed with phosphate buffered saline containing 0.01–0.1% Tween 20 (PBST). For tissue GFAP and αII-spectrin blots, membranes were incubated with horseradish peroxidase (HRP)-linked secondary antibodies and bands were detected with Clarity Western ECL (Bio-Rad, Hercules, CA, United States). Blots were visualized and quantified using the ImageQuant LAS4000 and ImageQuant TL v7.0 software (GE Healthcare, Pittsburgh, PA, United States). For calpain-II and GFAP and αII-spectrin blots of CSF, InfraRed (IR)-Dye labeled secondary antibodies were used to visualize bands on the Odyssey CLx imaging system (LI-COR Biosciences, Lincoln, NE, United States) and bands were quantified using ImageStudio v5.2 software (LI-COR Biosciences, Lincoln, NE, United States).

### GFAP electro-chemiluminescent enzyme-linked immunosorbent assay (ELISA)

Concentrated CSF samples were evaluated for GFAP content using an in-house assay developed for detection with the Mesoscale Discovery (MSD) platform (37). Samples and standards were loaded in duplicate. Samples were diluted in 1xPBS, pH 7.8 (Bio-Rad, Hercules, CA, United States) then incubated in plates manually coated with 25 μg/mL polyclonal anti-GFAP (ab7260, Abcam, Cambridge, MA, United States) in 1xPBS, pH 7.8. Plates were then incubated with mixed monoclonal anti-GFAP detection antibodies (BD556330, BD Biosciences, San Jose, CA, United States) and anti-mouse sulfo-tag antibody (MSD, Rockville, MD, United States) at 0.5 μg/mL each in 0.5% Blocker B prepared in 1xPBS, pH 7.8. Protein content was derived from standard curves using recombinant human GFAP protein in PBS (Banyan Biomarkers, Alachua, FL, United States; standard range: 0.156–10 ng/mL) fit by a cubic third order polynomial function. Values derived from PBS were used as blanks and subtracted from all samples. Analyte quantitation (ng/mL) was determined by electro-chemiluminescent signal with a Meso QuickPlex SQ120 (Meso Scale Discovery, Rockville, MD, United States).

### Data management and statistical analysis

For tissue western blots, mean target band densities for probe and PBBI groups are presented as a percent of the sham control value (100%) calculated from individual membranes. For CSF western blots, target bands at each time point were normalized to the mean value of a positive control band generated from frontal cortex tissue lysates collected 24 h post-PBBI. Since variable volumes of CSF were used to concentrate proteins, these normalized band values were subsequently normalized by the original μL of CSF loaded for protein concentration. Concentrations of GFAP (ng/mL) obtained in ELISA experiments were also normalized by the original μL of CSF. For CSF western blots and ELISAs, any negative values that were generated (i.e., band signal equal to less than the calculated background signal or sample signal less than blank) were set as ‘0’ for the purpose of data analysis. Sample sizes are detailed in the figure legends. Please note that 9–10 rats/group and time point were initiated on study, however, due to inability to collect CSF or insufficient collection volumes from all subjects, additional rats were injured for acute collections time points when necessary. All values are presented as mean ± SEM. Outliers as determined by ROUT analysis (*Q* = 0.1%) were excluded from all data sets. For neuroscore, western blotting experiments, and ELISAs, significant injury effects were determined at each time point using a one-way ANOVA followed by Tukey's multiple comparisons test to compare experimental groups. Two-tailed Pearson correlation coefficients (*r*) were determined using all available data points from Western blot experiments with sham, probe, and PBBI rats. For all analyses, results were considered statistically significant when *p* < 0.05. All statistical analyses were performed using GraphPad Prism v6 (La Jolla, CA, United States).

## Results

### Neurological impairment

**Figure 1:**
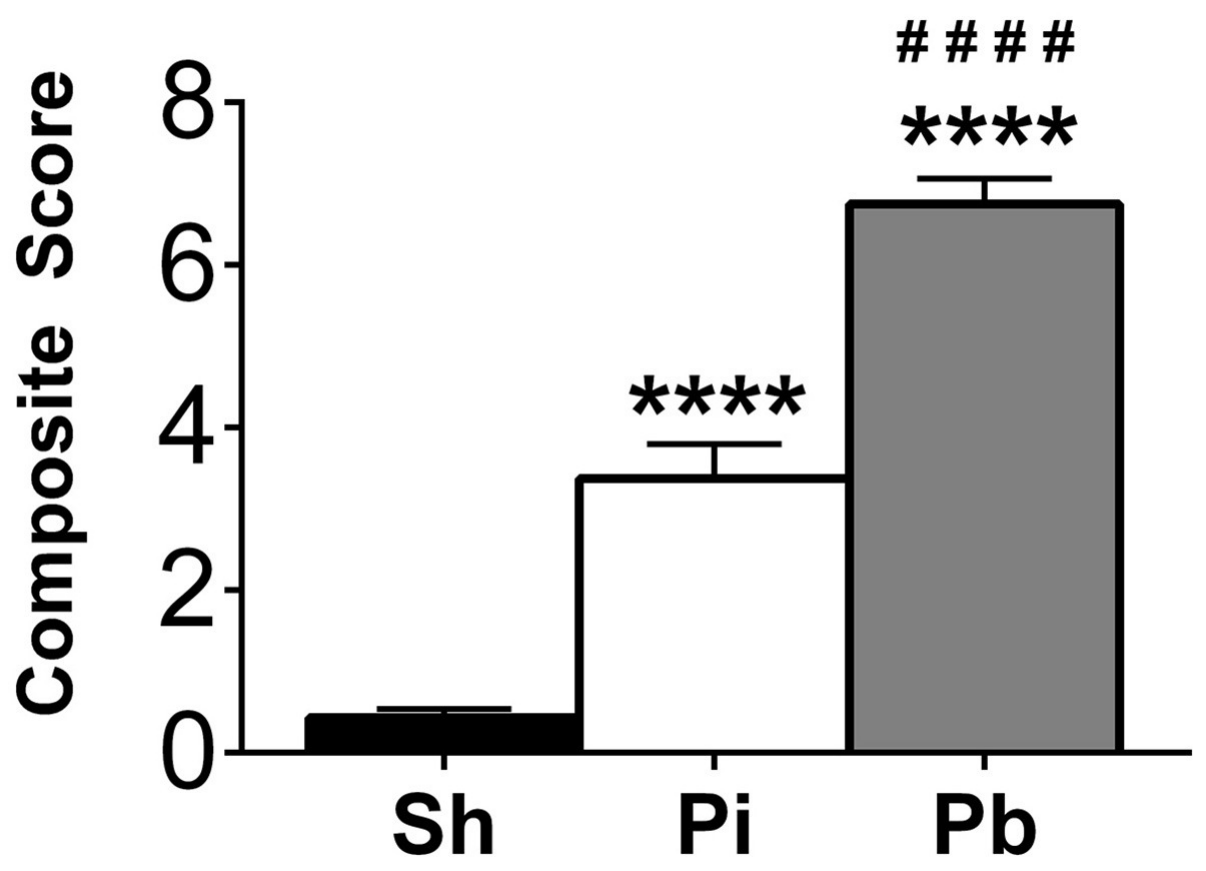
Penetrating brain injury results in significant acute neurological deficits. Neuroscore evaluations were performed 24 h following injury in Sham (Sh, black bar), Probe Injury (Pi, white bar), and PBBI (Pb, gray bar) groups. Composite values are presented here from each injury condition regardless of the tissue collection time point. *N* = 44 – 50 per group. ^*^^*^^*^^*^p ≤ 0.0001 Pi or Pb vs. Sh; ^*####*^*p* ≤ 0.0001 Pb vs. Pi, one-way ANOVA with Tukey's multiple comparisons test.

To evaluate neurological deficits following the probe injury and PBBI, neuroscore values were assessed at 24 h after injury compared to sham control procedures for all cohorts. Both probe injury (3.38 ± 0.42, range 0–12) and PBBI (6.75 ± 0.31, range 1.5–12) resulted in significantly elevated cumulative neuroscore values compared to sham (0.43 ± 0.10, range 0–1.5) (Figure 1), indicating that both injuries result in acute neurological impairment. Additionally, PBBI animals had significantly elevated neuroscore values over probe-injured rats.

### Quantitation of GFAP, αII-spectrin, and calpain-II in brain tissues

**Figure 2:**
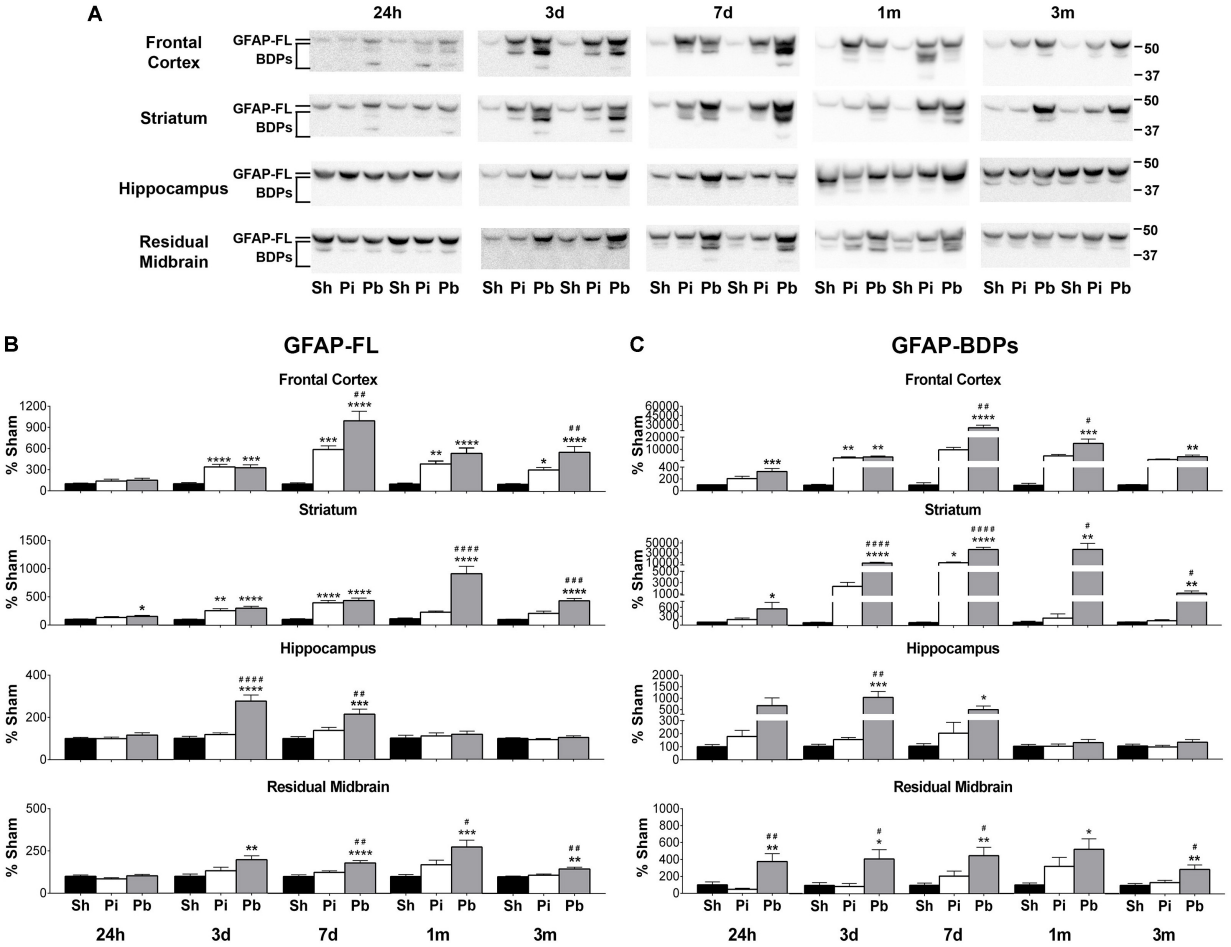
Penetrating brain injury results in robust longitudinal increases in GFAP and GFAP-BDPs in brain tissue. **(A)** Representative western blots to illustrate GFAP and its BDPs at 24 h, 3 days, 7 days, 1 month, and 3 months post injury or sham manipulation in the frontal cortex, striatum, hippocampus, and residual midbrain areas. Full length GFAP (GFAP-FL) was detected as indicated at 50 kDa, with breakdown products ranging from 37 to 48 kDa. Depicted gels were loaded in the order of Sham (Sh), Probe (Pi), PBBI (Pb) as indicated. Quantitation of **(B)** GFAP-FL at 50 kDa and **(C)** GFAP-BDPs at 37–48 kDa is presented as percent change from sham for each time point and brain region examined. Values are presented as mean ± SEM for Sh (black bars), Pi (white bars), or Pb (gray bars) groups. *N* = 9 – 10 per group and time point, ^*^p < 0.05, ^*^^*^p < 0.01, ^*^^*^^*^p < 0.001, ^*^^*^^*^^*^p ≤ 0.0001 Pi or Pb vs. Sh; ^#^*p* < 0.05, ^*##*^*p* < 0.01, ^*###*^*p* < 0.001, ^*####*^*p* ≤ 0.0001 Pb vs. Pi, one-way ANOVA with Tukey's multiple comparisons test. Full blot images are available in Supplementary Figures S3–S6.

To determine the abundance of both full length GFAP (GFAP-FL) at 50 kDa and its breakdown products (BDPs) from 37 to 48 kDa, the brain regions that directly encompass the injury trajectory (frontal cortex, striatum) and adjacent distal regions (hippocampus, residual midbrain) were examined by western blotting at 24 h, 3 days, 7 days, 1 month, and 3 months following injury (Figure 2A). Quantitative analysis indicated that GFAP-FL and GFAP-BDPs were elevated over time in both the probe injury and PBBI groups compared to sham. Probe injury alone resulted in significantly increased GFAP-FL in the brain regions which encompass the injury trajectory starting at 3 days (frontal cortex: peak of 589% at 7 days; striatum: peak of 397% at 7 days) (Figure 2B). This elevation was sustained through 3 months in the frontal cortex. PBBI resulted in a slight, but significant, increase in GFAP-FL in the striatum starting at 24 h. Beginning at 3 days, GFAP-FL was significantly increased following PBBI in all brain regions and persisted in the frontal cortex (peak of 995% at 7 days), striatum (peak of 912% at 1 month), and residual midbrain (peak of 276% at 1 month) through 3 months, whereas increases in the hippocampus (peak of 276% at 3 days) were resolved by 1 month.

As with GFAP-FL, significant elevations of GFAP-BDPs following probe injury compared to sham were detected only in the frontal cortex and striatum at 3 days (3247%) or 7 days (11,123%), respectively (Figure 2C). In contrast, PBBI robustly increased GFAP-BPDs at all time points in the frontal cortex (peak of 25,450% at 7 days), striatum (peak of 38,230% at 7 days), and residual midbrain regions (peak of 525% at 1 month). PBBI significantly increased GFAP-BDPs in the hippocampus at 3 days (1026%) and 7 days (485%). The consequence of increased injury severity on GFAP-FL and GFAP-BDPs was also determined by evaluating differential protein abundance between probe and PBBI injured rats. The abundance of GFAP-FL and GFAP-BDPs following PBBI was significantly increased over probe injury in a number of brain regions and time points assessed (Figures 2B,C), although this observation was less frequent than comparisons with sham.

**Figure 3:**
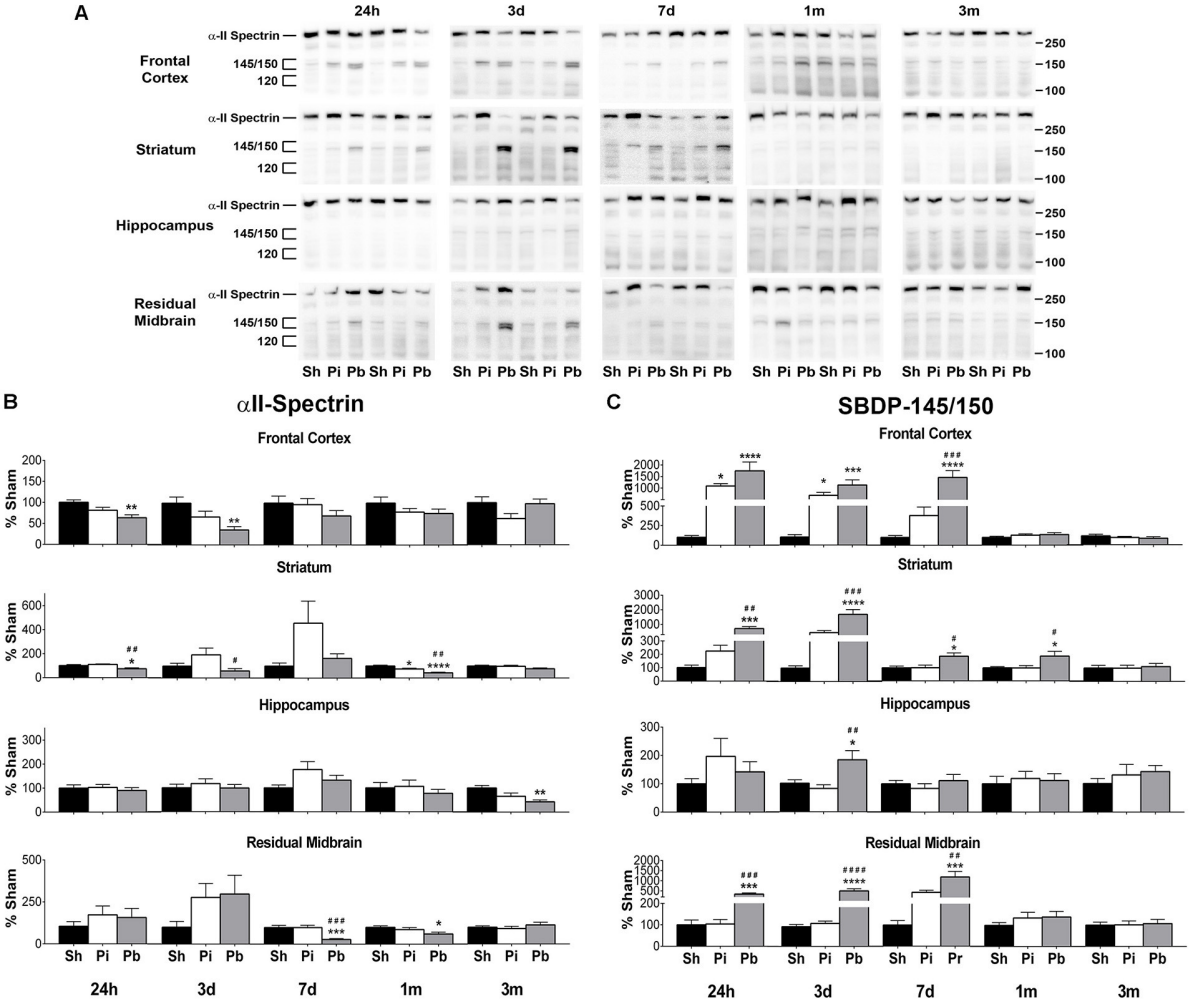
Penetrating brain injury reduces the abundance of αII-spectrin with concomitant increases in SBDP in brain tissues. **(A)** Representative western blots to illustrate αII-spectrin and SBDP at 24 h, 3 days, 7 days, 1 month, and 3 months post injury or sham manipulation in the frontal cortex, striatum, hippocampus, or residual midbrain regions. Alpha-II-spectrin was detected as indicated at 280 kDa, with SBDPs present at approximately 145/150 and 120 kDa. Depicted gels were loaded in the order of Sham (Sh), Probe (Pi), PBBI (Pb) as indicated. Quantitation of **(B)** αII-spectrin and **(C)** SBDP-145/150 is presented here as percent change from sham for each time point and brain region examined. Quantitation of SBDP-120 is available in Supplementary Figure S1. Values are presented as mean ± SEM for Sh (black bars), Pi (white bars), or Pb (gray bars) groups. *N* = 9 – 10 per group and time point, ^*^p < 0.05, ^*^^*^p < 0.01, ^*^^*^^*^p < 0.001, ^*^^*^^*^^*^p ≤ 0.0001 Pi or Pb vs. Sh; ^#^*p* < 0.05, ^*##*^*p* < 0.01, ^*###*^*p* < 0.001, ^*####*^*p* ≤ 0.0001 Pb vs. Pi, one-way ANOVA with Tukey's multiple comparisons test. Full blot images are available in Supplementary Figures S7–S10.

Next, the abundance of αII-spectrin and spectrin breakdown products (SBDPs) was assayed in the same manner as GFAP. Visual inspection of the western blots confirmed that full length αII-spectrin was detectable at 280 kDa as expected. After probe injury and PBBI, SBDP-145/150 was robustly evident, while SBDP-120 was minimally detected and poorly resolved (Figure 3A). Quantitative analysis revealed that probe injury decreased αII-spectrin compared to sham at 1 month in the striatum only (−24%) (Figure 3B). PBBI acutely reduced αII-spectrin in the areas of the immediate injury trajectory (frontal cortex: maximum decrease of −64% at 3 days, striatum: −26% at 24 h only) compared to sham. In distal injury areas, no acute reductions in αII-spectrin were observed compared to sham. However, PBBI lowered αII-spectrin levels at subacute and chronic time points (hippocampus: −57% at 3 month only, residual midbrain: maximum decrease of −72% at 7 days).

Concomitant increases in SBDP-145/150 were also observed (Figure 3C). Probe injury increased SBDP-145/150 in the frontal cortex at 24 h and 3 days (peak of 1091% at 24 h). Following PBBI, SBDP-145/150 increased starting at 24 h and remained elevated through 7 days in the frontal cortex (peak of 1742% at 24 h), striatum (peak of 1731% at 3 days), and residual midbrain (peak of 1230% at 7 days). Levels detected in the striatum remained elevated until 1 month. In the hippocampus, PBBI led SBDP-145/150 to increase at 3 days, only (182%). Quantitative analysis of SBDP-120 revealed that this fragment was elevated, albeit at a much smaller magnitude than observed with SBDP-145/150 (Supplementary Figure S1). Probe injury increased SBDP-120 at 3 month in the frontal cortex only (262%). PBBI resulted in significantly increased SBDP-120 in the frontal cortex at 24 h and 3 days (peak of 357% at 24 h) and delayed increases in the hippocampus at 3 months (434%) and the residual midbrain at 7 days and 1 month (peak of 349% at 1 month). Additionally, PBBI altered levels of αII-spectrin, SBDP-145/150, and SBDP-120 compared to probe insertion alone.

**Figure 4:**
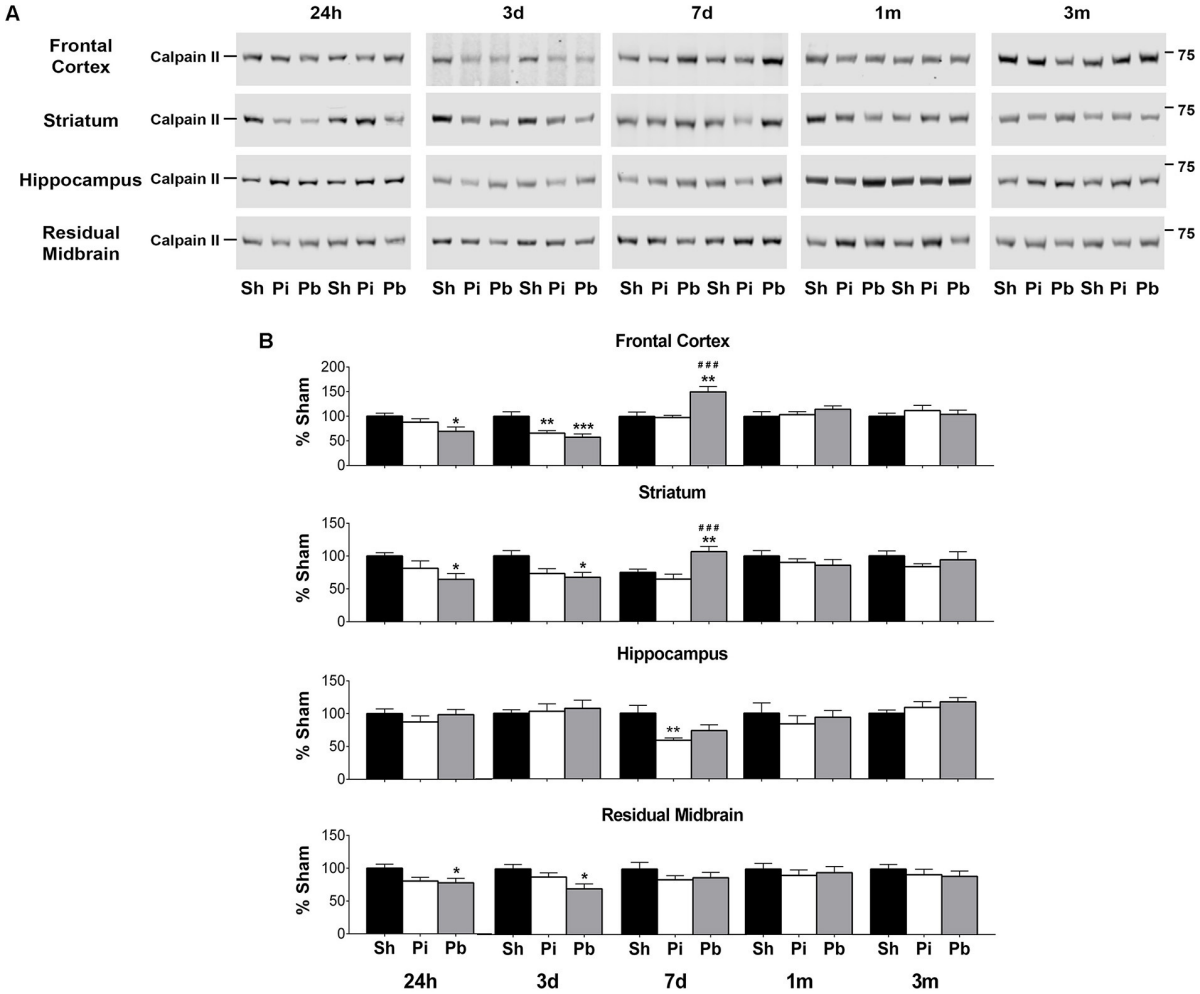
Penetrating brain injury alters the abundance of full length calpain-II in brain tissues. **(A)** Representative western blots to illustrate full length calpain-II at 24 h, 3 days, 7 days, 1 month, and 3 months post injury or sham manipulation in the frontal cortex, striatum, hippocampus, or residual midbrain. Full length calpain-II was detected at approximately 72 kDa. Depicted gels were loaded in the order of Sham (Sh), Probe (Pi), PBBI (Pb) as indicated. **(B)** Quantitation of calpain-II is presented as percent change from sham for each time point and brain region examined. Values are presented as mean ± SEM for Sh (black bars), Pi (white bars), or Pb (gray bars). *N* = 9 – 10 per group and time point, ^*^p < 0.05, ^*^^*^p < 0.01, ^*^^*^^*^p < 0.001 Pi or Pb vs. Sh; ^*###*^*p* < 0.001 Pb vs. Pi, one-way ANOVA with Tukey's multiple comparisons test. Full blot images are available in Supplementary Figures S11–S14.

To determine if calpain-II was differentially abundant after brain trauma, protein levels were assessed by western blot in the cohorts used to define GFAP, αII-spectrin, and respective BDP levels (Figure 4). Prominent bands corresponding to full-length calpain-II were detected at 73 kDa (Figure 4A). Compared to sham, probe injury reduced calpain-II abundance in the frontal cortex (−34% at 3 days) and the hippocampus (−51% at 7 days) (Figure 4B). PBBI acutely reduced calpain-II abundance in the frontal cortex (maximum decrease of −44% at 3 days), striatum (maximum decrease of −34% at 24 h), and residual midbrain (maximum decrease of −30% at 3 days) compared to sham. Surprisingly, at 7 days after PBBI, increased calpain-II was observed in the frontal cortex and striatum (150 and 142% respectively) compared to both sham and probe-injured rats.

### Detection of GFAP, GFAP-BDPs and SBDPs in CSF

**Figure 5:**
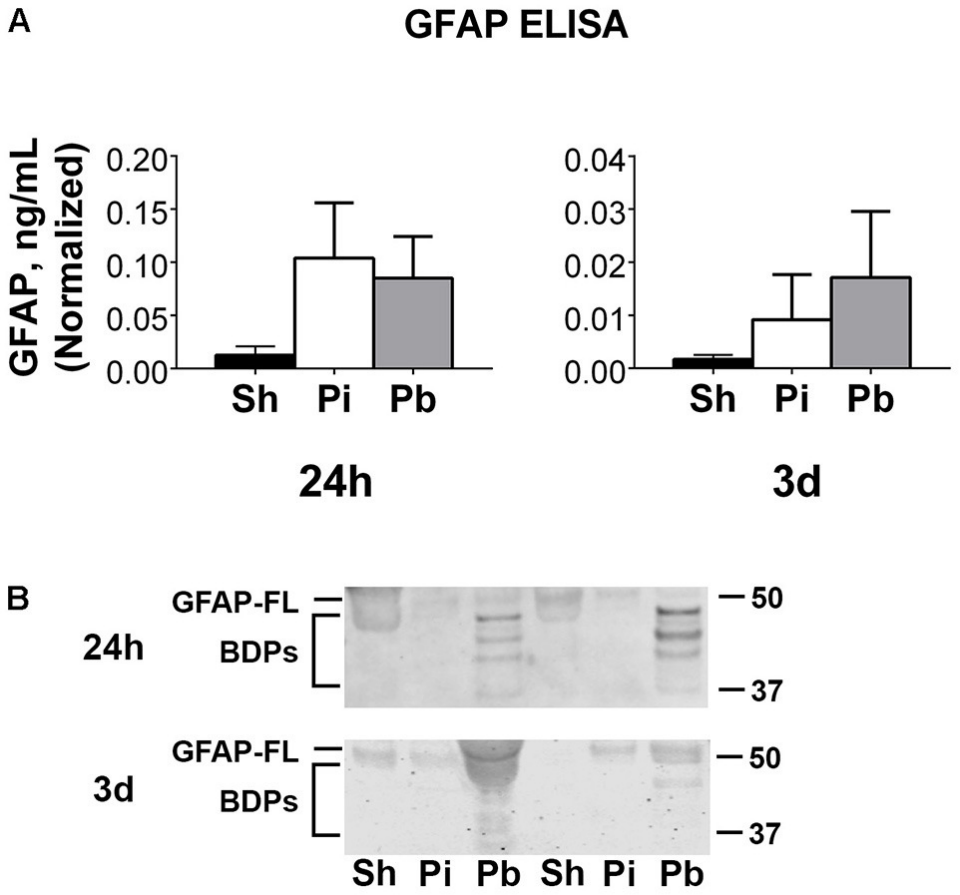
Full length GFAP and its BDPs are detectable acutely in CSF following penetrating brain injury. **(A)** Enzyme-linked immunosorbent assays (ELISA) for GFAP were performed on CSF samples collected at 24 h or 3 days after injury or sham control procedures. Data is quantified as ng/mL normalized to the μL of CSF available for sample processing as described in materials and methods. Values are presented as mean ± SEM for Sh (black bars), Pi (white bars), or Pb (gray bars) groups. **(B)** Representative western blots to assess the CSF samples presented in **(A)** for GFAP-FL and GFAP BDPs. GFAP-FL was detected at 50 kDa while BDPs were detected from 37 to 48 kDa. *N* = 6 – 13 per group and time point. Full blot images are available in Supplementary Figure S15.

Cerebral spinal fluid samples were used to determine the abundance of GFAP, αII-spectrin, and their BDPs after injury. Total GFAP ELISAs (representing both GFAP-FL and GFAP-BDPs) indicated that neither probe nor PBBI resulted in significantly altered total GFAP at 24 h or 3 days after injury (Figure 5A). Total GFAP in CSF collected 7 days through 3 months was not readily detectable (data not shown). To discern if either GFAP-FL or GFAP-BDPs, rather than total GFAP, may change significantly as a result of injury in these samples, western blots were performed (Figure 5B). Visual inspection revealed the presence of GFAP-FL and the characteristic banding pattern for GFAP-BDPs at 24 h and 3 days. Quantitation of this data revealed no significant alteration in GFAP-FL and GFAP-BDPs as determined by western blot (Supplementary Figure S2). As with ELISA analysis, levels in most samples were not readily detectable (data not shown) although GFAP-FL and GFAP-BDPs were evident in certain animals from 7 days to 3 month time points. Interestingly, GFAP-BDPs were detectable in a limited number of sham animals at acute through chronic times after injury.

**Figure 6:**
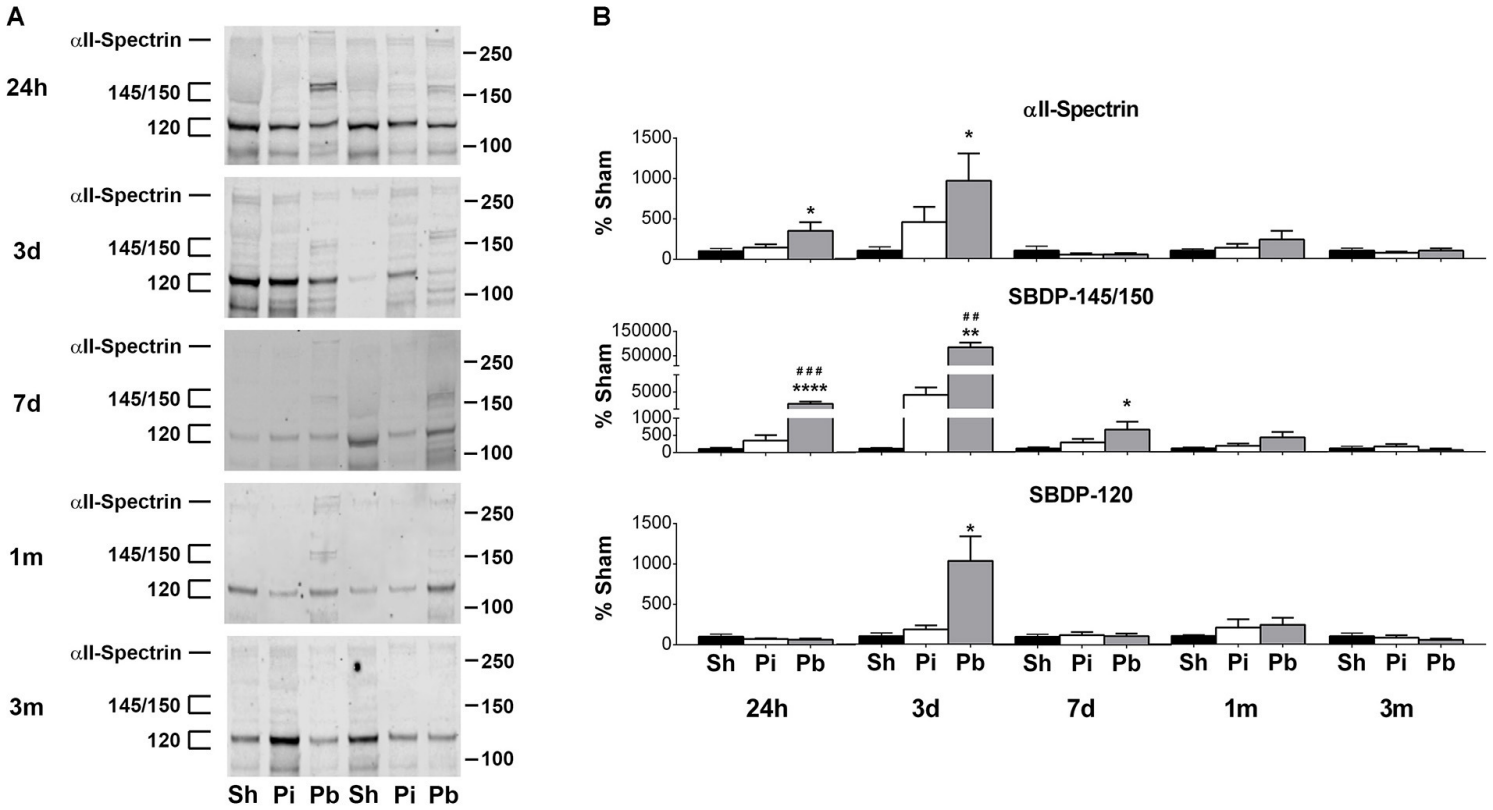
Alpha-II-spectrin and SBDP-145/150 and SBDP-120 are detectable longitudinally in CSF following penetrating brain injury. **(A)** Representative western blots in CSF collected at 24 h, 3 days, 7days, 1 month, and 3 months after injury or sham control manipulations. Alpha-II-spectrin was detected at 280 kDa while SBDPs were detected at 145/150 and 120 kDa. Gels were loaded in the order of Sham (Sh), Probe (Pi), PBBI (Pb) as indicated. **(B)** Quantitation of αII-spectrin and SBDPs at the indicated molecular weights is presented here as band density normalized to original μL of CSF available for sample processing as described in materials and methods. Values are presented as mean ± SEM for Sh (black bars), Pi (white bars), or Pb (gray bars) groups. *N* = 7 – 13 per group and time point, ^*^p < 0.05, ^*^^*^p < 0.01, ^*^^*^^*^^*^p ≤ 0.0001 Pi or Pb vs. Sh; ^*##*^*p* < 0.01, ^*###*^*p* < 0.001, Pb vs. Pi, one-way ANOVA with Tukey's multiple comparisons test. Full blot images are available in Supplementary Figure S16.

Western blotting of CSF indicated that αII-spectrin, SBDP-145/150, and SBDP-120 were present as late as 3 months after injury (Figure 6A). Quantitative analysis demonstrated that while probe injury did not alter αII-spectrin or SBDP levels, PBBI resulted in significant elevation of these analytes compared to both sham and probe injured groups (Figure 6B). Alpha II-spectrin was increased to 352 and 967% at 24 h and 3 days, respectively, while SBDP-145/150 was elevated by 2248, 84,068%, and by 649% at 24 h, 3 days, and 7 days post-PBBI, respectively. SBDP-120 rose by 1035% at 3 days.

### Correlations with protein levels in brain tissue regions

To determine if CSF levels of GFAP, αII-spectrin, and their BDPs may have utility to predict abundance of the same protein levels in brain tissues, correlation analyses were performed using grouped data points from all three conditions as described in materials and methods. These correlations between specific protein levels in CSF and the corresponding protein levels in brain tissues were performed for each brain region and time point. GFAP-BDPs, but not GFAP-FL, revealed many significant positive correlations between CSF and tissues (Supplementary Table S1). GFAP-FL in CSF correlated with levels detected in the hippocampus at 3 days alone (*r* = 0.579). Starting at 24 h, GFAP-BDPs in CSF correlated with these same cleavage products in the frontal cortex, striatum, and residual midbrain (*r* range: from 0.476 to 0.569). This observation persisted at 3 days with the frontal cortex, striatum, and hippocampus (*r* range: 0.511–0.797). Similar to the results for GFAP, the relationship between SBDP, but not αII-spectrin, in CSF and brain tissues was significant in many instances (Table 1). This result was overwhelmingly true for SBDP-145/150 where significant correlations between CSF and brain tissue abundance were observed at 24 h in the striatum and residual midbrain (*r* = 0.582 and 0.655, respectively) and at 3d in the striatum, hippocampus, and residual midbrain (*r* range: 0.498–0.918). This correlation persisted at 7 days in the frontal cortex, striatum, and residual midbrain areas (*r* range: 0.507–0.777). CSF SBDP-120 was positively associated with values detected in the frontal cortex at 7 days post injury only (*r* = 0.560).

**Table 1:** Correlation results between full length αII-spectrin or SBDP protein levels in CSF and tissues specified by brain region and time point.

	**24 h**		**3 days**		**7 days**		**1 month**		**3 months**	
	***r***	***p-*value**	***r***	***p-*value**	***r***	***p-*value**	***r***	***p-*value**	***r***	***p-*value**
*αII-Spectrin*										
FCX	−0.338	0.078	−0.417	0.096	0.141	0.483	0.273	0.153	−0.338	0.079
STM	−0.315	0.102	−0.172	0.509	−0.082	0.691	−0.145	0.454	−0.221	0.249
HC	−0.017	0.931	−0.256	0.322	0.049	0.808	−0.169	0.380	−0.030	0.881
RMB	−0.229	0.251	0.470	0.057	0.075	0.717	0.168	0.384	0.014	0.942
*SBDP-145/150*										
FCX	0.287	0.139	0.295	0.206	0.777	< 0.0001^****^	−0.065	0.738	0.398	0.066
STM	0.582	0.001^**^	0.880	< 0.0001^****^	0.507	0.008^**^	0.124	0.523	0.251	0.249
HC	0.095	0.631	0.498	0.030^*^	−0.188	0.348	−0.219	0.254	−0.016	0.943
RMB	0.655	0.000^***^	0.918	< 0.0001^****^	0.559	0.002^**^	0.081	0.678	−0.179	0.413
*SBDP-120*										
FCX	−0.083	0.673	−0.094	0.693	0.560	0.002^**^	−0.188	0.358	−0.070	0.733
STM	0.155	0.450	0.105	0.660	−0.059	0.776	−0.299	0.138	0.247	0.235
HC	−0.240	0.229	0.030	0.902	−0.361	0.064	0.059	0.775	−0.200	0.317
RMB	−0.359	0.066	−0.241	0.307	0.181	0.365	−0.047	0.821	−0.073	0.731

Pearson r correlation analyses were performed between levels of αII-spectrin or SBDPs in CSF and brain tissues by region and time point post injury. Protein levels were determined by Western blot at each time point post injury and were expressed as normalized values for CSF markers and as a percent sham for tissue markers for analysis. Sham, Probe, and PBBI injured rats were all included for analysis. Significant correlations are marked with

* *(p < 0.05)*,

** *(p < 0.01)*,

*** (p < 0.001), or

**** *(p ≤ 0.0001)*.

*CSF, cerebrospinal fluid; FCX, frontal cortex; STM, striatum; HC, hippocampus; RMB, residual midbrain*.

Additional correlations were performed to determine the relationship between acute neurological deficits and brain tissue protein abundance grouped from all three injury conditions. Overall, acute neuroscore values were positively associated with GFAP-FL and GFAP-BDPs in brain (Table 2). At 3 days (*r* range: 0.498–0.749) and 7 days (0.476–0.828), GFAP-FL and GFAP-BDP abundance in all brain regions correlated significantly with neurological deficits. Acute neuroscore values and brain αII-spectrin levels were negatively associated with one another throughout acute-chronic injury (Table 3). This negative association was strongest at 7 days (*r* = −0.632 in residual midbrain). In accordance with the gain of SBDPs in brain tissues, analysis indicated that these levels were positively associated with acute neurological deficits. This was overwhelmingly true of SBDP-145/150 detected at 24 h–7 days, wherein this correlation was significant among all four brain regions at 3 days, but was greatest with the residual midbrain (*r* = 0.742). Fewer instances of significance were observed with analysis of SBDP-120 levels, where the strongest correlation was with the residual midbrain at 7 days (*r* = 0.664).

**Table 2:** Correlation results between GFAP-FL and GFAP-BDP protein tissue levels and neuroscore values specified by brain region and time point.

	**24 h**		**3 days**		**7 days**		**1 month**		**3 months**	
	***r***	***p-*value**	***r***	***p-*value**	***r***	***p-*value**	***r***	***p-*value**	***r***	***p-*value**
*GFAP-FL*										
FCX	0.293	0.116	0.498	0.005^**^	0.749	< 0.0001^****^	0.587	0.007^**^	0.392	0.036^*^
STM	0.482	0.007^**^	0.521	0.003^**^	0.559	0.002^**^	0.677	0.002^**^	0.361	0.054
HC	0.243	0.196	0.731	< 0.0001^****^	0.647	0.000^***^	−0.001	0.995	0.109	0.573
RMB	0.104	0.586	0.695	< 0.0001^****^	0.665	< 0.0001^****^	0.601	0.005^**^	0.250	0.190
*GFAP-BDPs*										
FCX	0.655	< 0.0001^****^	0.528	0.003^**^	0.800	< 0.0001^****^	0.431	0.074	0.272	0.162
STM	0.630	0.000^***^	0.698	< 0.0001^****^	0.828	< 0.0001^****^	0.467	0.044^*^	0.357	0.058
HC	0.264	0.202	0.476	0.010^*^	0.404	0.027^*^	0.105	0.659	0.236	0.217
RMB	0.427	0.021^*^	0.701	< 0.0001^****^	0.594	0.001^***^	0.553	0.012^*^	0.424	0.024^*^

Pearson r correlation analyses were performed between neuroscore and levels of GFAP-FL and GFAP-BDPs in brain tissues by region and time point post injury. Neuroscore assessment was performed at 24 h after injury. Protein levels were determined by Western blot and were expressed as percent sham. Sham, Probe, and PBBI injured rats were all included for analysis. Significant correlations are marked with

* *(p < 0.05)*,

** *(p < 0.01)*,

*** (p < 0.001), or

**** *(p ≤ 0.0001). FCX, frontal cortex; STM, striatum; HC, hippocampus; RMB, residual midbrain*.

**Table 3:** Correlation results between αII-spectrin and SBDP protein tissue levels and neuroscore values specified by brain region and time point.

	**24 h**		**3 days**		**7 days**		**1 month**		**3 months**	
	***r***	***p-*value**	***r***	***p-*value**	***r***	***p-*value**	***r***	***p-*value**	***r***	***p-*value**
*αII-Spectrin*										
FCX	−0.470	0.009^**^	−0.568	0.001^**^	−0.274	0.143	−0.290	0.214	−0.133	0.499
STM	−0.353	0.055	−0.327	0.078	−0.123	0.525	−0.520	0.019^*^	−0.095	0.623
HC	−0.041	0.831	−0.169	0.374	0.159	0.401	−0.058	0.807	−0.465	0.013^*^
RMB	0.160	0.408	0.228	0.226	−0.632	0.000^***^	−0.412	0.071	0.431	0.020^*^
*SBDP-145/150*										
FCX	0.681	< 0.0001^****^	0.565	0.001^**^	0.692	< 0.0001^****^	0.136	0.567	−0.243	0.213
STM	0.628	0.000^***^	0.590	0.001^***^	0.731	< 0.0001^****^	0.293	0.211	−0.246	0.198
HC	0.039	0.837	0.434	0.019^*^	0.123	0.518	0.266	0.257	0.499	0.007^**^
RMB	0.517	0.004^**^	0.742	< 0.0001^****^	0.715	< 0.0001^****^	0.554	0.011^*^	0.196	0.307
*SBDP-120*										
FCX	0.410	0.025^*^	0.456	0.011^*^	0.249	0.184	0.146	0.540	0.115	0.560
STM	−0.134	0.497	0.037	0.844	−0.086	0.658	0.133	0.575	−0.351	0.073
HC	0.208	0.278	0.054	0.776	−0.125	0.511	−0.090	0.713	0.347	0.065
RMB	0.031	0.872	−0.278	0.138	0.664	< 0.0001^****^	0.267	0.255	−0.273	0.169

Pearson r correlation analyses were performed between neuroscore and levels of αII-spectrin and SBDPs in brain tissues by region and time point post injury. Neuroscore assessment was performed at 24 h after injury. Protein levels were determined by Western blot and were expressed as percent sham. Sham, Probe, and PBBI injured rats were all included for analysis. Significant correlations are marked with

* *(p < 0.05)*,

** *(p < 0.01)*,

*** (p < 0.001), or

**** *(p ≤ 0.0001). FCX, frontal cortex; STM, striatum; HC, hippocampus; RMB, residual midbrain*.

## Discussion

**Figure 7:**
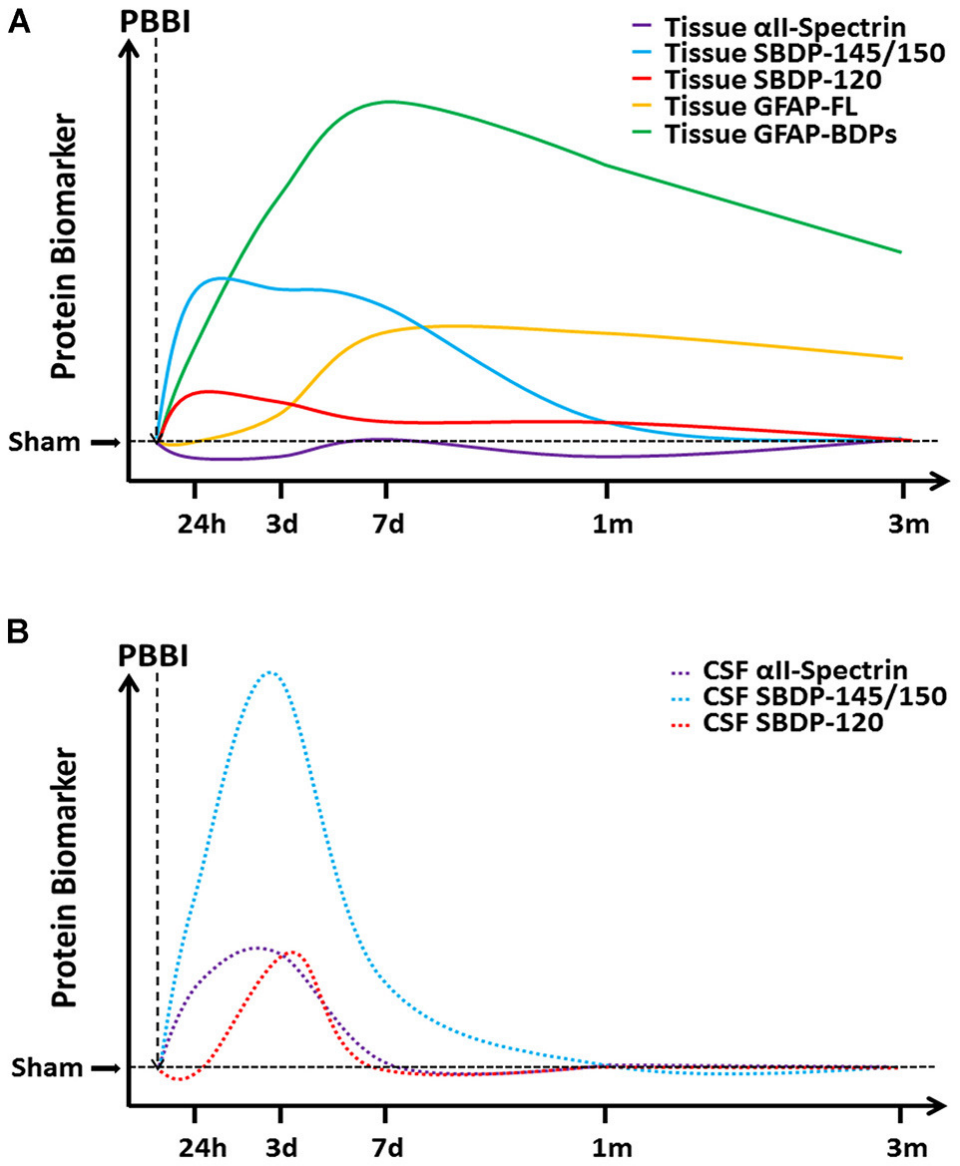
A cartoon timeline of protein based biomarkers following PBBI. Changes in protein biomarker levels are presented here for **(A)** tissue proteins and **(B)** CSF proteins as indicated, with increasing time on the *x*-axis and increasing protein abundance on the *y*-axis. Please note that axes are not drawn to scale and that these curves have been approximated from PBBI rats, with tissue curves representing changes in the brain regions of the immediate injury trajectory (frontal cortex and striatum).

This study provides a comprehensive evaluation of well-described TBI biomarkers (GFAP, αII-spectrin, and their BDPs) in brain tissue regions and CSF throughout acute-chronic (24 h to 3 months) injury progression. A representation of this timeline following PBBI is indicated for brain tissues (Figure 7A) as approximated from the immediate areas of the primary injury trajectory (frontal cortex and striatum) and CSF (Figure 7B). PBBI resulted in widespread time-dependent changes in protein abundance, which was especially robust for BDPs as opposed to intact, full-length counterparts within several brain regions. While effects were also evident to some degree in animals exposed to insertion of the probe alone, the majority of injury effects appear to result from the ballistic-like force of the injury captured by the rapid inflation/deflation of the balloon inside the rat brain. This study indicated that CSF SBDP-145/150 levels may have utility during acute-subacute injury to serve as an indicator of neuronal degradation in the brain. Further, this study indicated that acute neurological deficits were associated with levels of GFAP, αII-spectrin, and BDPs as late as 3 months after injury.

### Temporal progression of astroglial and neuronal BDPs

GFAP and αII-spectrin proteolysis and subsequent BDP generation in brain tissue is well-established following TBI but has focused on the acute time frame (24–27). This study indicates that GFAP and αII-spectrin degradation, and the resulting increased generation of BDPs, is progressive and dominant during subacute–chronic time frames. The increase in GFAP-BDPs and SBDPs after both probe injury and PBBI provide evidence that both GFAP-FL in astrocytes and αII-spectrin in neurons undergo long-term proteolysis as a consequence of PBBI, albeit with distinct temporal profiles. These diverging profiles indicate that reactive astrogliosis (GFAP-FL) and astroglial protein degradation (GFAP-BDP) are comparatively delayed following PBBI and persist chronically. In contrast, neuronal cytoskeletal protein degradation (SBDP-145/150) is prominent within acute-subacute time frames but resolves afterward, which is likely due to the resolution of the core lesion into an intracranial cavity by 7 days following PBBI (38). This pattern may extend to other injury models as well, since acute elevation of SBDP within 7–14 days (39) and chronic upregulation of GFAP up to 1 year (40, 41) have been previously reported. These distinct temporal profiles associated with GFAP and αII-spectrin inform the duration of drug treatment paradigms targeted toward specific mechanisms, such as the chronic sustainment of astrogliosis and proteolysis of GFAP-FL into GFAP-BDPs.

Calpain- or caspase- mediated protein degradation is proposed to be a key factor in TBI mediated neurodegeneration. Generation of GFAP-BDPs and SBDP-145/150 are products of calpain-mediated proteolysis (26), whereas proteolysis by caspase-3 activity results primarily in generation of SBDP-120 (42). The results presented here indicate the predominance of calpain-mediated cleavage products in the acute–chronic periods following PBBI. Additionally, this study demonstrated an overall loss in full length calpain-II, an indirect marker of calpain activation (43), following PBBI within brain regions that were proximal as well as somewhat distal to the injury tract. Calpain-II is decreased during truncation and activation prior to degradation of protein substrates (42), however, calpain-II fragments that represent the active form of the enzyme were not detectable in this study. As a minor point, full-length calpain-II increased 7 days after PBBI, a finding that has previously been reported in a blast model of TBI (44) and may reflect a compensatory response to injury that has yet to be clearly defined based on TBI mechanism and/or severity.

### Variation across multiple brain regions

This study indicates that chronic, progressive degradation occurs not only in brain regions containing the primary lesion as previously reported (30, 32, 45) but also within areas that are adjacent to the primary wound, albeit with differing magnitudes. In this study, the effects of PBBI were not only more severe in the immediate regions of the primary injury trajectory (i.e., frontal cortex) as opposed to more distal areas (i.e., hippocampus), but also appeared more quickly and persisted for a longer duration. Interestingly, the temporal profiles of GFAP-FL and GFAP-BDPs revealed that peak levels varied with time and between regions containing the primary injury lesion and distal injury areas. These divergent temporal profiles likely reflect a more rapid resolution of secondary injury effects in the less severely injured areas, whereas astrogliosis and proteolysis continue to increase progressively past 3 days before improving somewhat by 3 months in the more severely injured brain regions. Distinct profiles of protein abundance by brain region have been reported with SBDP-145/150 following CCI (39) and the data presented here are consistent with previous work following PBBI showing delayed injury induced responses in the thalamus compared to the primary lesion (38).

This work corroborates and expands upon previous work from our laboratory, which defined the presence of GFAP, αII-spectrin, and their BDPs in a coronal brain section containing the primary injury lesion through 7 days after PBBI (27) with a few noteworthy exceptions. First, our previous data indicated that GFAP-FL abundance declines at 3 days after injury, which contrasts with the elevations observed in this study. Second, levels of αII-spectrin were nearly ablated by 3 days following PBBI previously, whereas here we see reductions of a smaller magnitude. Finally, significantly increased SBDP-145/150 were not seen previously at 24 h after PBBI as opposed to the robust increases in most brain regions assessed at this time-point in the current study. Whereas the first study isolated an ipsilateral coronal brain section combining both cortical and subcortical structures in each analysis, this study dissected specific ipsilateral brain regions with differing anatomical locations in relation to the primary injury trajectory. These data indicate that differential protein abundance of these tissue markers varies greatly between these distinct brain regions; thus, a greater resolution within each region of interest is likely to account for the differences observed here compared to our previous work. In addition, evaluating specific regions of interest provides useful information regarding regional injury progression as opposed to examination of a single brain section containing the primary injury lesion (19, 46).

### The role of the ballistic-like injury component on magnitude and distribution of neurodegeneration

The differential effects of penetrating injury alone compared to a penetrating ballistic-like injury were examined here by inclusion of both the probe injury and PBBI groups, respectively. While both injuries resulted in acute neurological deficits compared to sham rats, these deficits were of a greater magnitude following PBBI compared to probe injury. Greater elevation in the tissue abundance of GFAP, αII-spectrin, and their BDPs in PBBI compared to probe injured rats was also observed longitudinally across multiple brain regions. This is consistent with previous work that has indicated elevated SBDP-145/150 following more severe CCI (47). These data provide both functional and molecular outcome measures to better distinguish how these different injuries may alter the progression of molecular pathology. For example, while significant changes in GFAP, αII-spectrin, and their BDPs following probe injury were observed, these instances were limited to the frontal cortex and striatum only with no evidence for the spread of this pathology to distal brain regions as seen following PBBI. This finding indicates that while probe insertion alone causes localized injury effects, the temporary cavity mimicking energy dissipation from a high-velocity bullet round plays a critical role in the severity of injury and the propagation of PBBI pathology to areas beyond the primary injury trajectory.

### Biofluid based biomarkers as direct correlates of tissue protein abundance

The identification and validation of biofluid based biomarkers for use in informing individualized treatment plans and/or monitoring the efficacy of treatment over time holds great potential for improving TBI patient outcomes. Both GFAP-BPDs (48) and SBDPs (49) have been reported in CSF samples taken from acute and subacute human TBI patients. These studies indicate that acute levels of CSF SBDP-145/150 are elevated in severe TBI patients who fare worse across multiple outcome metrics, including acute Glasgow Coma Scale and chronic Glasgow Outcome Scale scores. The results presented in this study suggest that SBDP-145/150, rather than GFAP, GFAP-BDPs, or SBDP-120, may have utility as a monitoring biomarker through subacute-chronic stages following injury. Additionally, multiple correlations between CSF GFAP-BDPs and SBDP-145/150 and tissue levels of these same proteins were significant. These data suggest that CSF levels of these BDPs may reflect their presence in brain tissues following injury. While some studies have assessed serum GFAP-BPDs specifically (50), others have evaluated a combination of both GFAP and GFAP-BDPs as biomarkers (51, 52). Our data demonstrated that CSF BDPs of GFAP and αII-spectrin, rather than their full length counterparts, were overwhelmingly correlated to these same protein forms in tissues, indicating that future studies evaluating the efficacy of CSF TBI biomarkers may benefit from measuring BDPs specifically. Whether these CSF biomarkers precede, co-occur, or follow injury-induced changes in these tissue proteins is unclear from our data. Interestingly, even in the absence of significant group effects, numerous significant correlations of acute and subacute levels of GFAP-BDPs and SBDP-145/150 between CSF and tissues were obtained, emphasizing the importance of these CSF biomarkers to predict molecular changes in individual subjects.

### Neurological deficits as predictors of protein degradation

Acute neurological deficits correlated significantly with acute-chronic brain tissue levels of GFAP, αII-spectrin, and BDPs, however, these associations presented in a different pattern than the CSF correlations discussed above. While significant CSF correlations resulted primarily from GFAP-BDPs and SBDP-145/150, significant relationships between neuroscore values and brain tissue protein levels were more generalized and not as limited to BDPs specifically. This was especially true for GFAP. One explanation for this difference is that more severe injuries that cause greater neurological deficits are more likely to result in generalized injury induced alterations in protein abundance. Thus, in clinical practice, the assessment of neurological deficits with rating scales such as the NOS-TBI may have more benefit for classification of injury severity (53) and prediction of extended outcomes (54) rather than predictive ability for specific chronic neurodegeneration.

## Conclusion

This study is the first to characterize acute through chronic profiles of the well-known TBI biomarkers GFAP, αII-spectrin, and their BDPs in distinct brain regions of interest, identify the presence of these proteins and their BDPs in CSF, and demonstrate significant correlations between the levels of BDPs present in CSF and brain tissues following PBBI in rats. These results indicate that a sustained period of reactive astrogliosis but finite period of axonal cytoskeletal degradation follow penetrating brain injury and that the detection of calpain generated BDPs in CSF may predict the underlying presence of these tissue proteins in real time. Additionally, the evidence for chronic proteolysis and astrogliosis presented in this study informs the design of drug treatment paradigms following penetrating TBI, indicating that prolonged treatments into subacute-chronic periods should be considered.

## Data availability statement

The raw data supporting the conclusions of this manuscript will be made available by the authors, without undue reservation, to any qualified researcher.

## Author contributions

KD, CC, AB, and DS designed the experiments and prepared the manuscript. KD, CC, and HH performed the experiments and conducted data analysis.

### Conflict of interest statement

The authors declare that the research was conducted in the absence of any commercial or financial relationships that could be construed as a potential conflict of interest.
