# Supplementary material for: Cerebrospinal Fluid Biomarkers Are Associated With Glial Fibrillary Acidic Protein and αII-spectrin Breakdown Products in Brain Tissues Following Penetrating Ballistic-Like Brain Injury in Rats
Source: Front Neurol. 2018 Jul 4;9:490. doi: 10.3389/fneur.2018.00490 (PMC6039567; doi:10.3389/fneur.2018.00490)
Supplement: Supplementary file 1 [file Data_Sheet_1.pdf]

Supplementary Material

Cerebrospinal Fluid Biomarkers are Associated with Glial Fibrillary Acidic Protein and αII-spectrin Breakdown Products in Brain Tissues Following Penetrating Ballistic-like Brain Injury in Rats

Kristen E. DeDominicis*, Hye Hwang, Casandra M. Cartagena, Deborah A. Shear, Angela M. Boutté

*** Correspondence:** Kristen DeDominicis, kristen.dedominicis@gmail.com

1 Supplementary Figures and Tables

1.1 Supplementary Figures

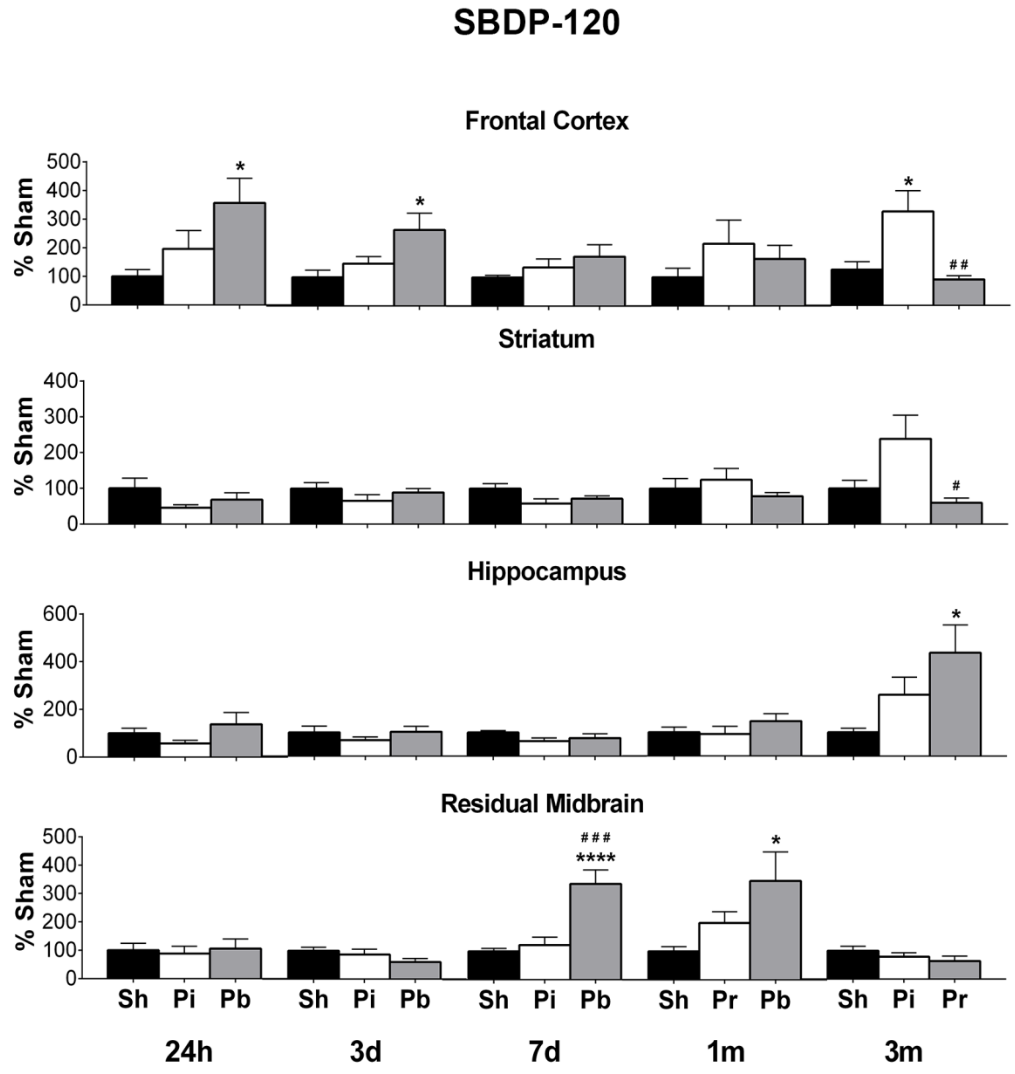

Supplementary Figure 1: Quantitation of SBDP-120 in brain tissue regions of interest. Representative western blot images are depicted in Figure 3 from Sham (Sh), Probe (Pi), and PBBI (Pb) rats at 24h, 3d, 7d, 1m, and 3m post injury in the frontal cortex, striatum, hippocampus, or residual midbrain regions. SBDP-120 is quantified as the percent change from Sh. Data is presented as mean ± SEM for Sh (black bars), Pi (white bars), or Pb (gray bars) groups. N = 9 - 10 per group and time point, * $p < 0.05$, **** $p \leq 0.0001$ Pi or Pb vs Sh; # $p < 0.05$, ## $p < 0.01$, ### $p < 0.001$, Pb vs Pi, One-way ANOVA with Tukey's multiple comparisons test.

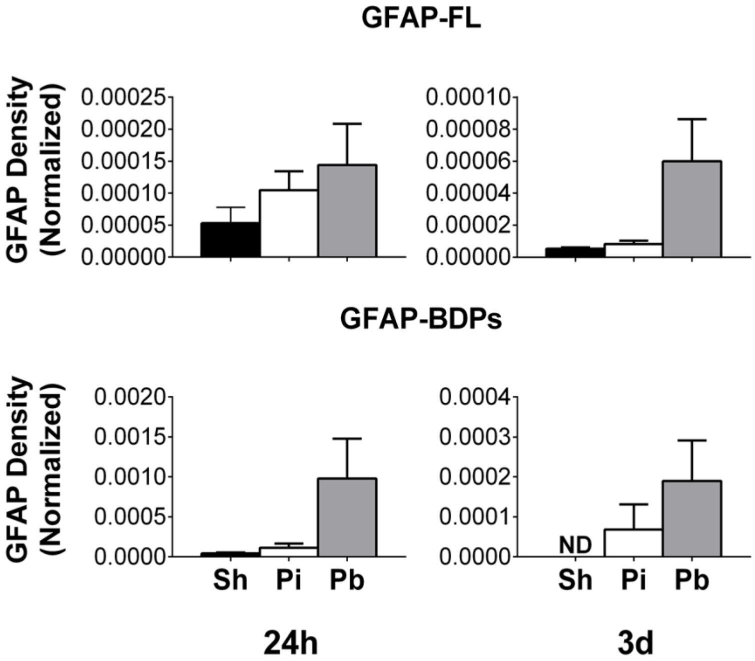

Supplementary Figure 2: Quantitation of GFAP-FL and GFAP-BDPs in CSF. Representative western blot images are depicted in Figure 5 from Sham (Sh), Probe (Pi), and PBBI (Pb) rats at 24h or 3d in CSF. GFAP-FL at 50kDa and BDPs at 37–48kDa are quantified as band density normalized to original volume (µL) of CSF available for sample processing as described in materials and methods. Values are presented as mean ± SEM for Sh (black bars), Pi (white bars), or Pb (gray bars) groups. N = 6 - 13 per group and time point. ND = not detectable.

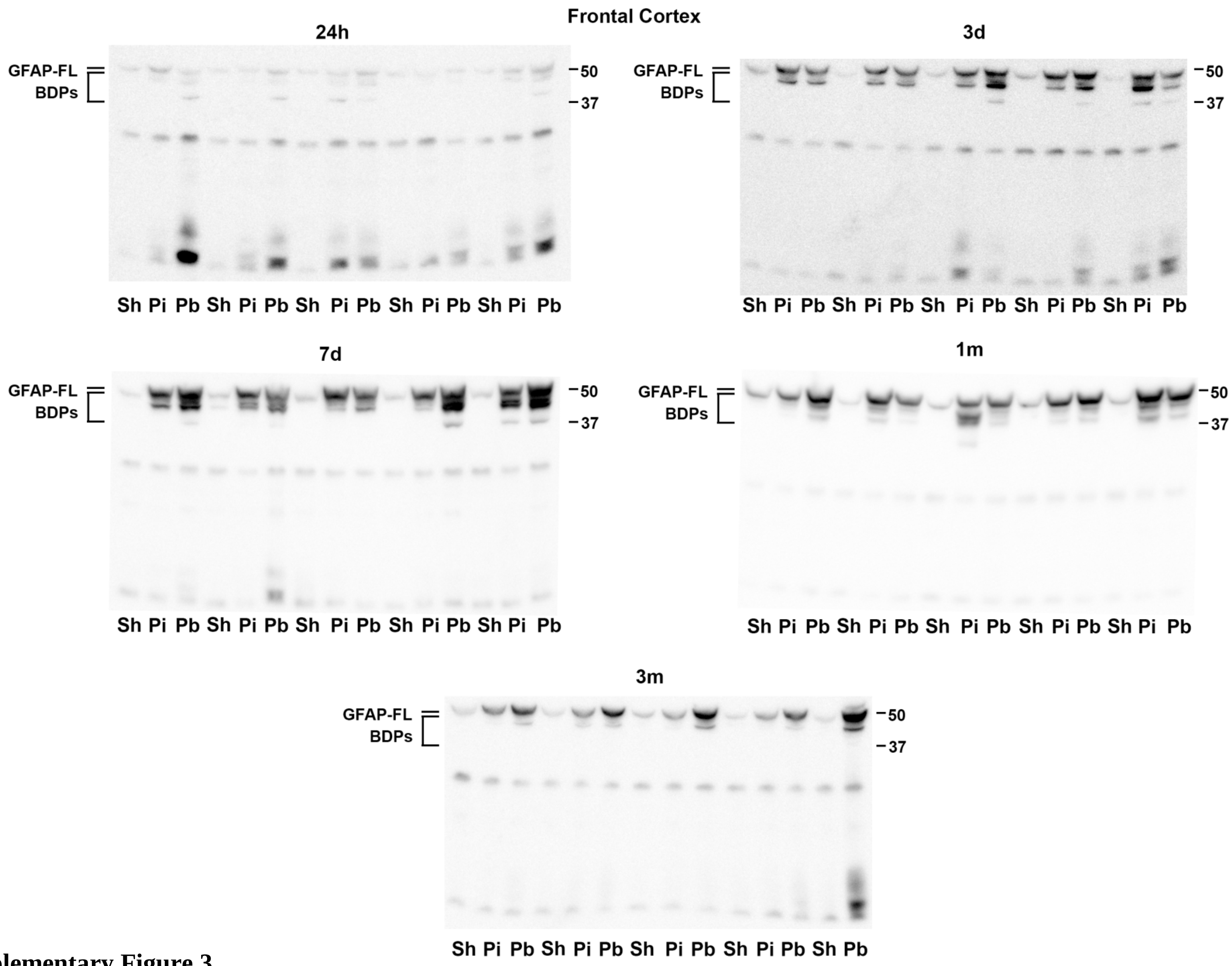

Supplementary Figure 3

Supplementary Figure 3: Full gel images of western blots for GFAP and GFAP-BDPs in frontal cortex from 24h – 3m, which were presented as cropped representative images in Figure 2. Lanes were loaded with Sham (Sh), Probe (Pi), or PBBI (Pb) samples as indicated.

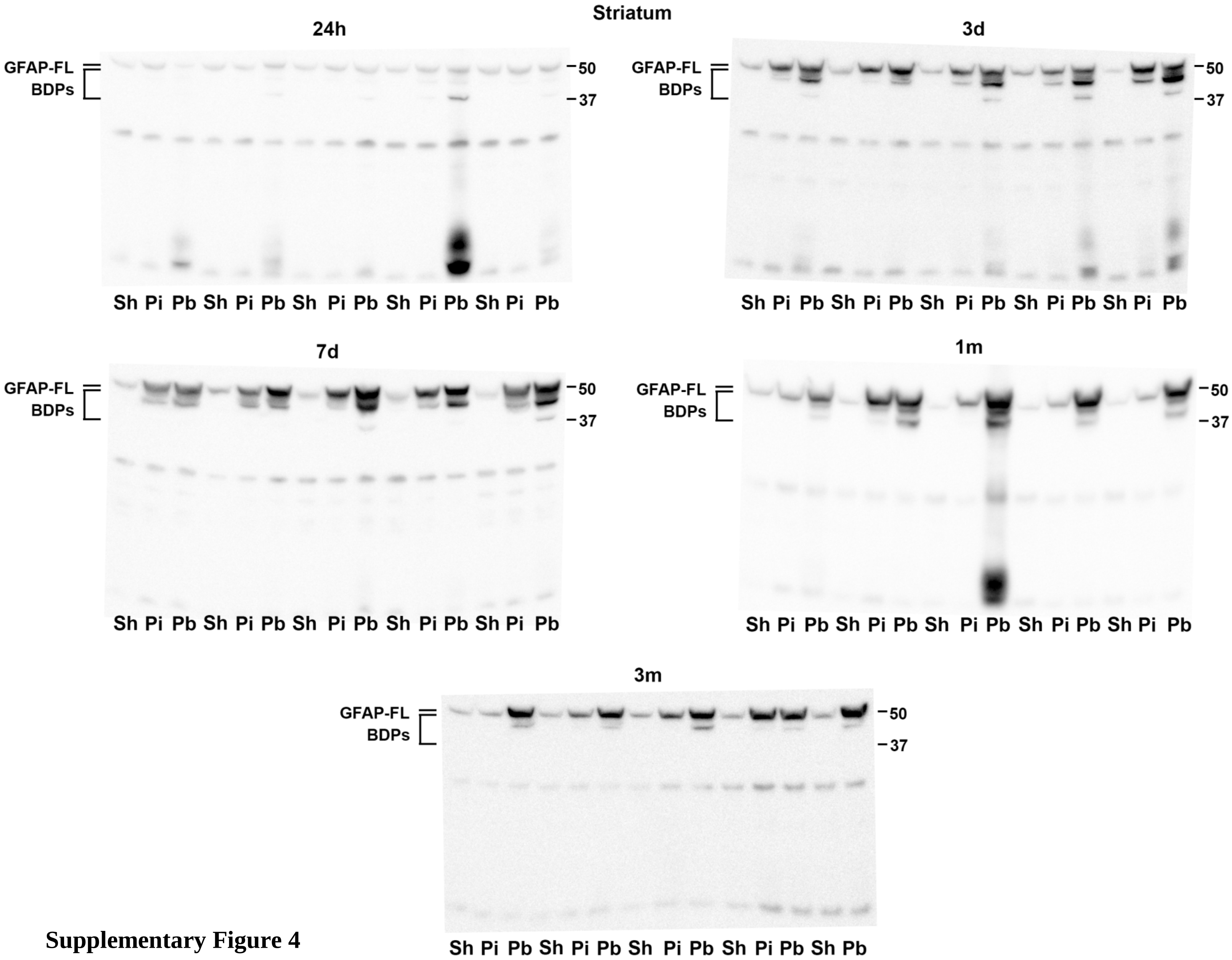

Supplementary Figure 4

Supplementary Figure 4: Full gel images of western blots for GFAP and GFAP-BDPs in striatum from 24h – 3m, which were presented as cropped representative images in Figure 2. Lanes were loaded with Sham (Sh), Probe (Pi), or PBBI (Pb) samples as indicated.

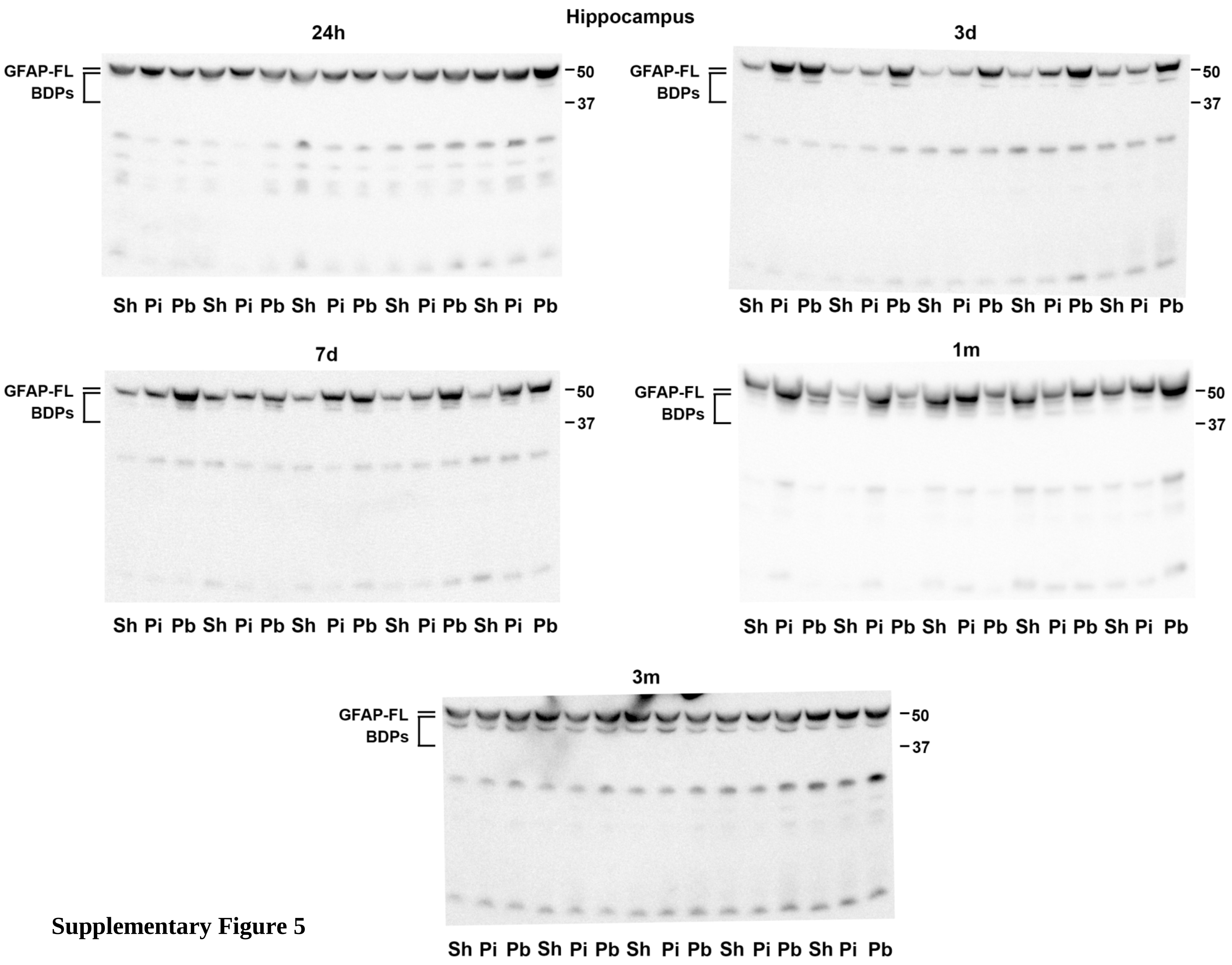

Supplementary Figure 5

Supplementary Figure 5: Full gel images of western blots for GFAP and GFAP-BDPs in hippocampus from 24h – 3m, which were presented as cropped representative images in Figure 2. Lanes were loaded with Sham (Sh), Probe (Pi), or PBBI (Pb) samples as indicated.

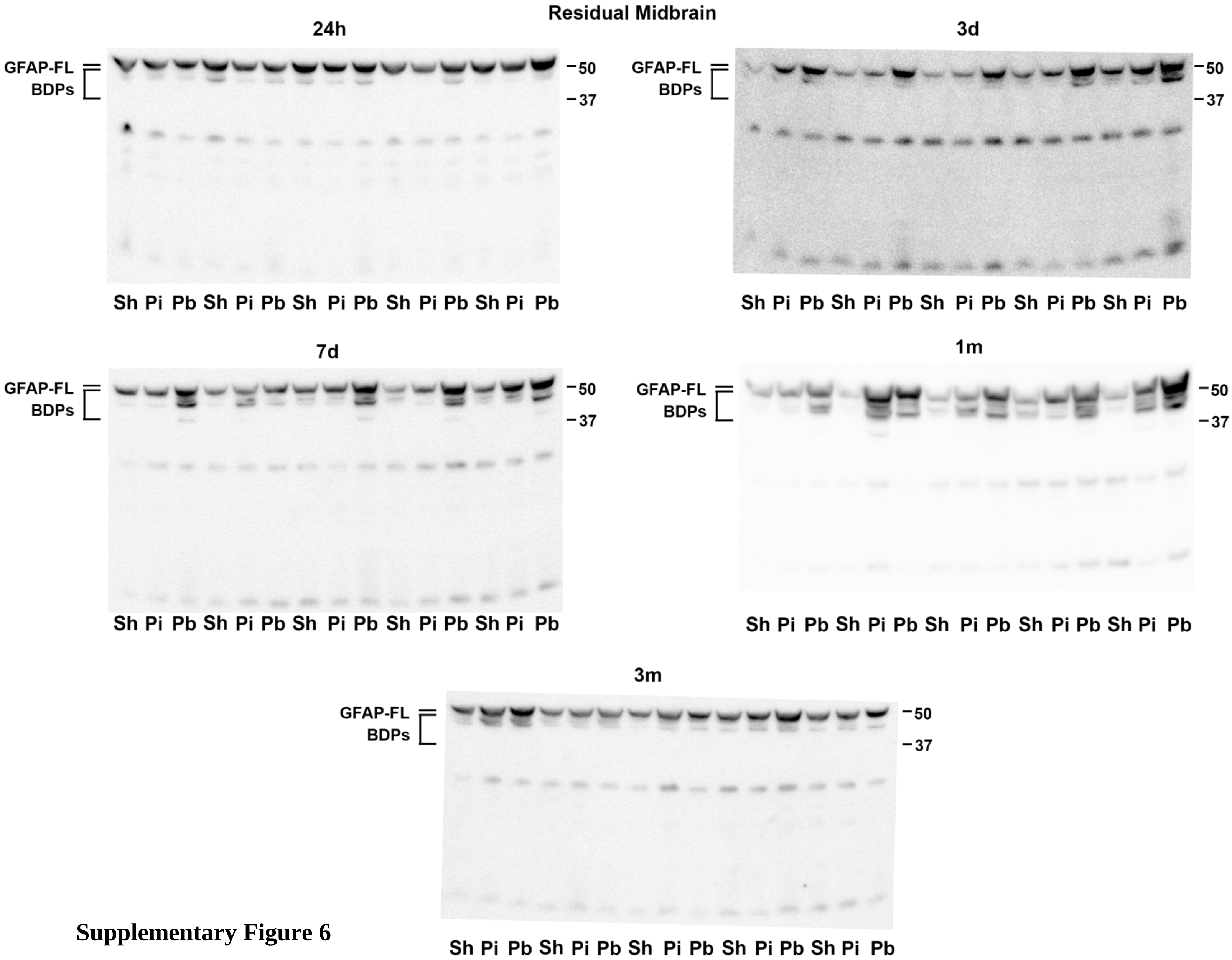

Supplementary Figure 6

Supplementary Figure 6: Full gel images of western blots for GFAP and GFAP-BDPs in residual midbrain from 24h – 3m, which were presented as cropped representative images in Figure 2. Lanes were loaded with Sham (Sh), Probe (Pi), or PBBI (Pb) samples as indicated.

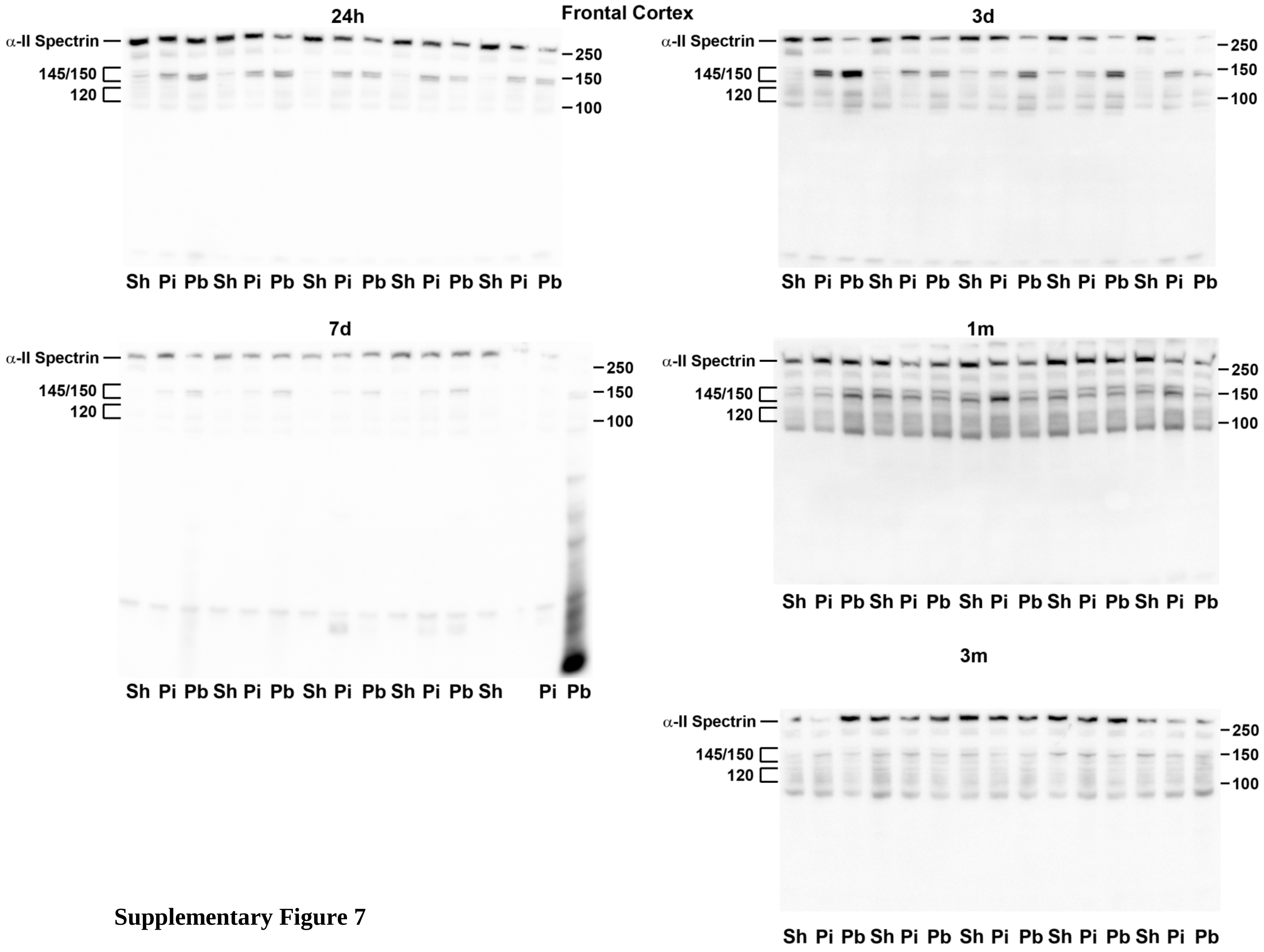

Supplementary Figure 7

Supplementary Figure 7: Full gel images of western blots for αII-spectrin and SBDPs in frontal cortex from 24h – 3m, which were presented as cropped representative images in Figure 3. Lanes were loaded with Sham (Sh), Probe (Pi), or PBBI (Pb) samples as indicated.

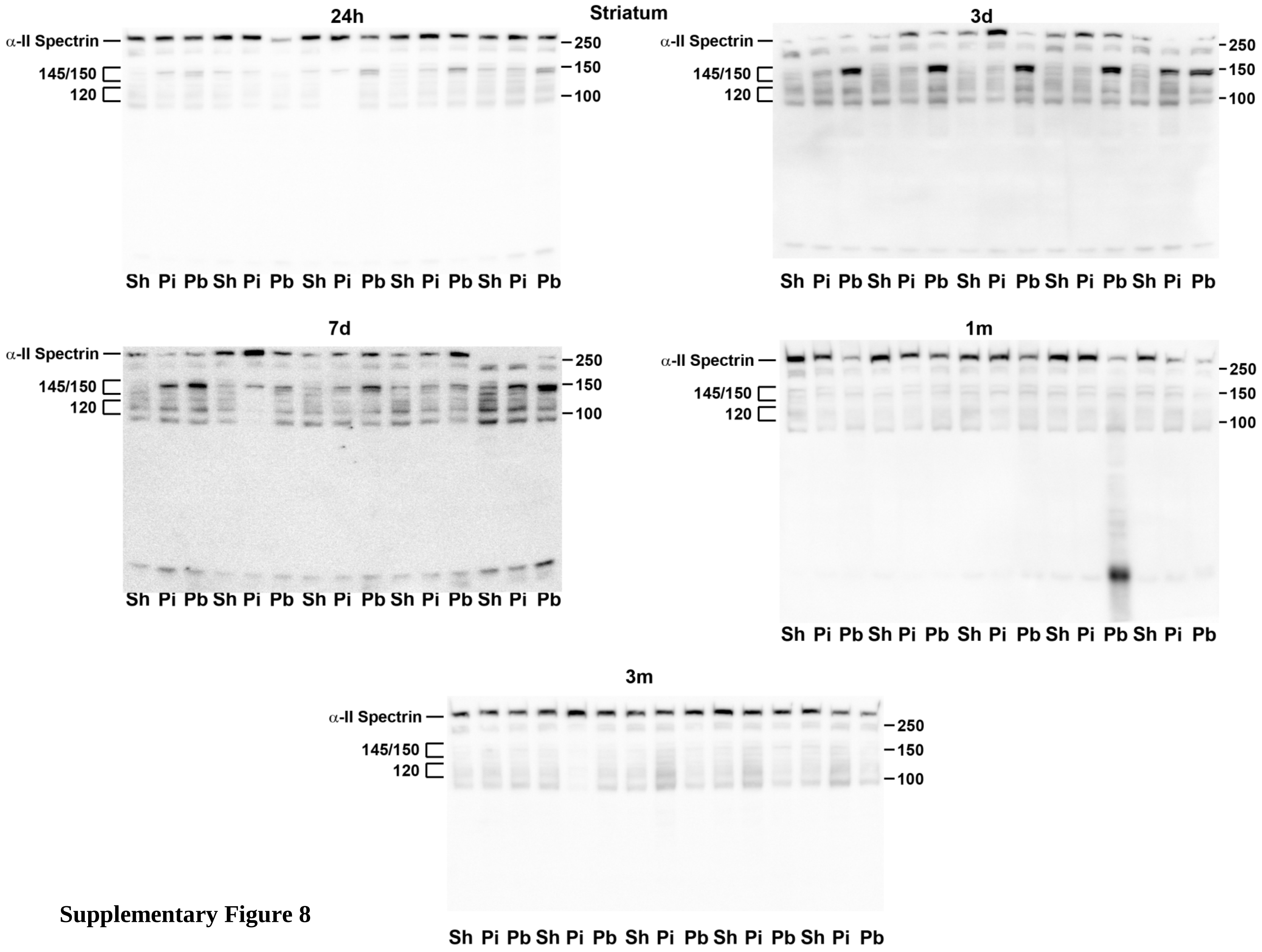

Supplementary Figure 8

Supplementary Figure 8: Full gel images of western blots for αII-spectrin and SBDPs in striatum from 24h – 3m, which were presented as cropped representative images in Figure 3. Lanes were loaded with Sham (Sh), Probe (Pi), or PBBI (Pb) samples as indicated.

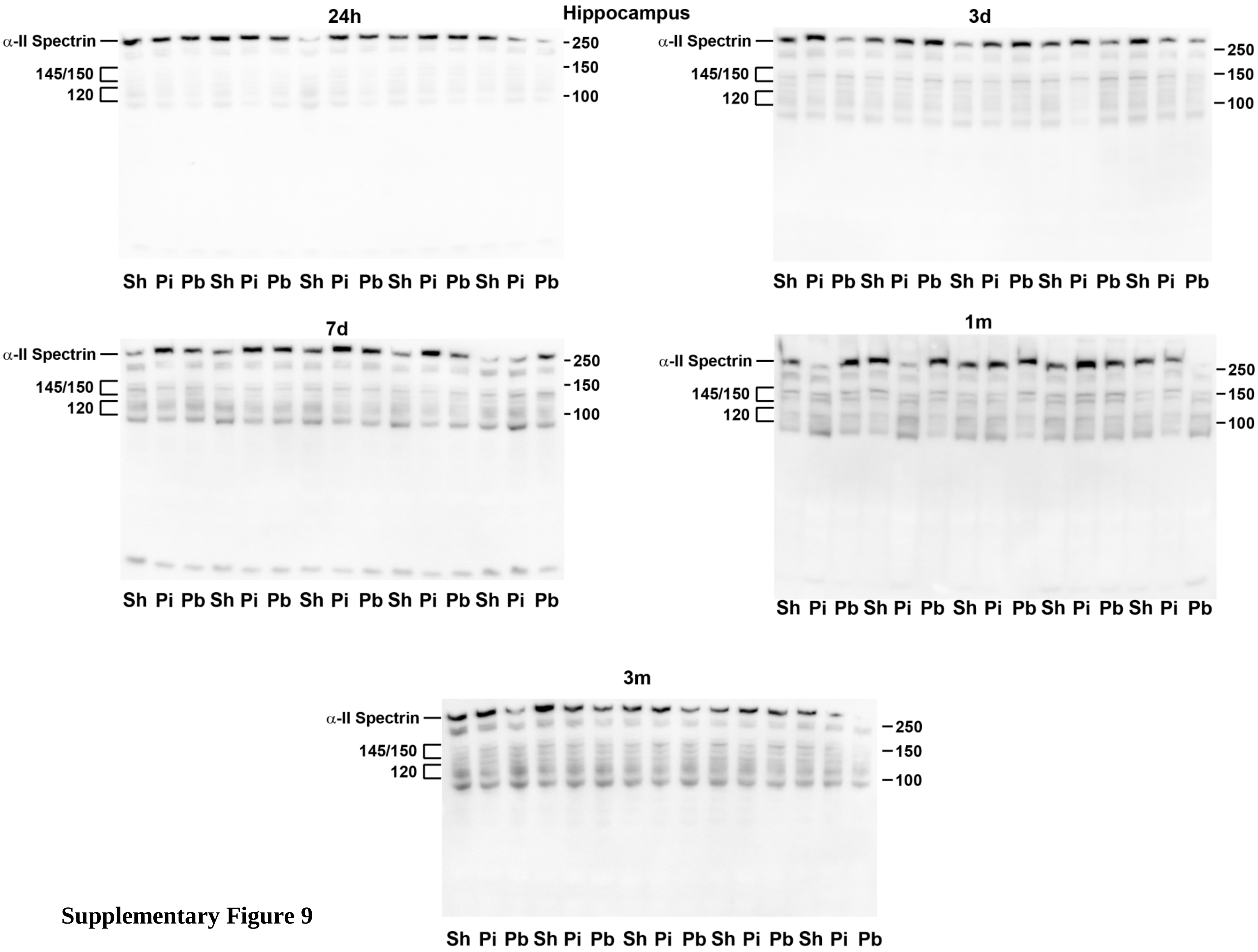

Supplementary Figure 9

Supplementary Figure 9: Full gel images of western blots for αII-spectrin and SBDPs in hippocampus from 24h – 3m, which were presented as cropped representative images in Figure 3. Lanes were loaded with Sham (Sh), Probe (Pi), or PBBI (Pb) samples as indicated.

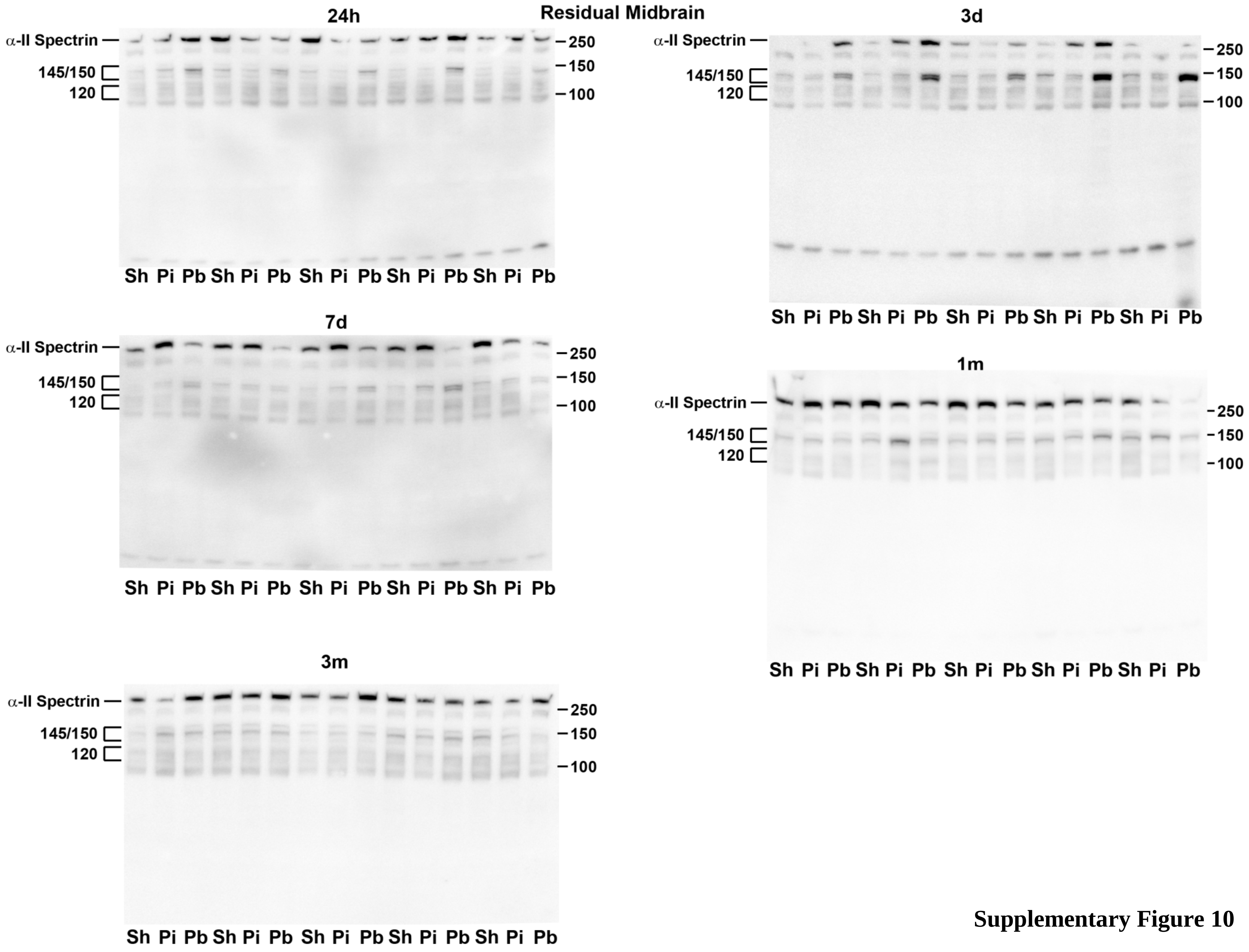

Supplementary Figure 10

Supplementary Figure 10: Full gel images of western blots for αII-spectrin and SBDPs in residual midbrain from 24h – 3m, which were presented as cropped representative images in Figure 3. Lanes were loaded with Sham (Sh), Probe (Pi), or PBBI (Pb) samples as indicated.

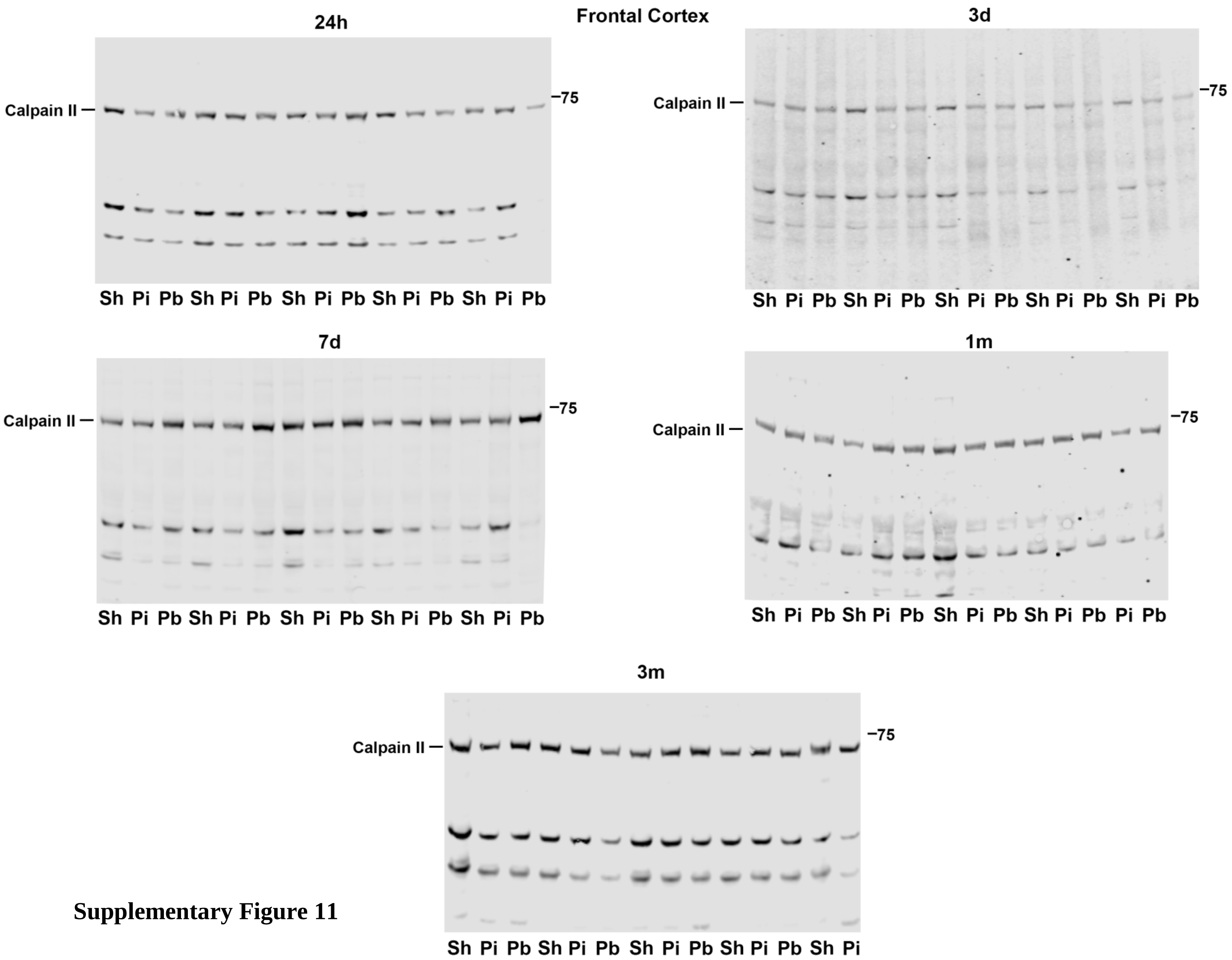

Supplementary Figure 11

Supplementary Figure 11: Full gel images of western blots for calpain-II in frontal cortex from 24h – 3m, which were presented as cropped representative images in Figure 4. Lanes were loaded with Sham (Sh), Probe (Pi), or PBBI (Pb) samples as indicated.

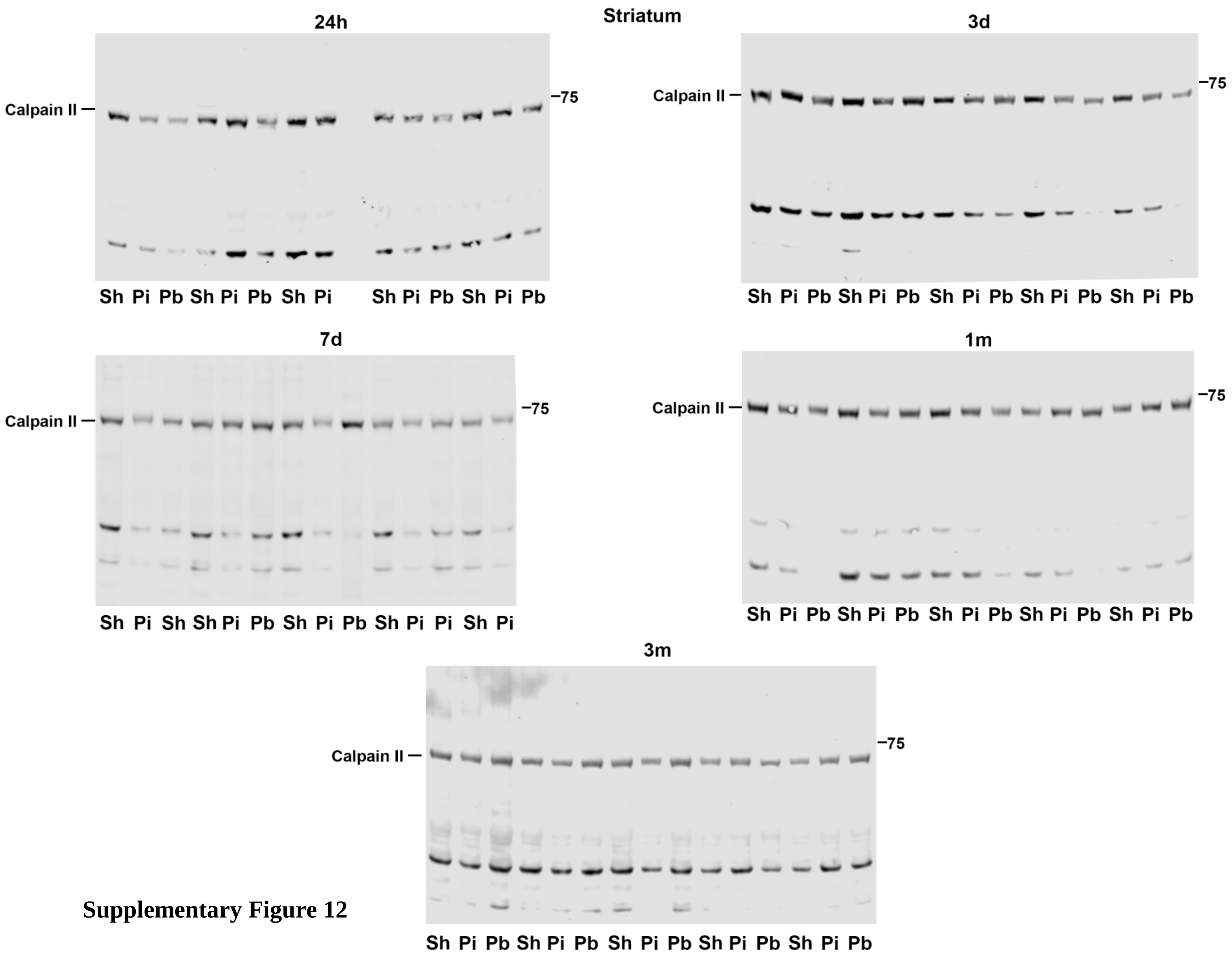

Supplementary Figure 12

Supplementary Figure 12: Full gel images of western blots for calpain-II in striatum from 24h – 3m, which were presented as cropped representative images in Figure 4. Lanes were loaded with Sham (Sh), Probe (Pi), or PBBI (Pb) samples as indicated.

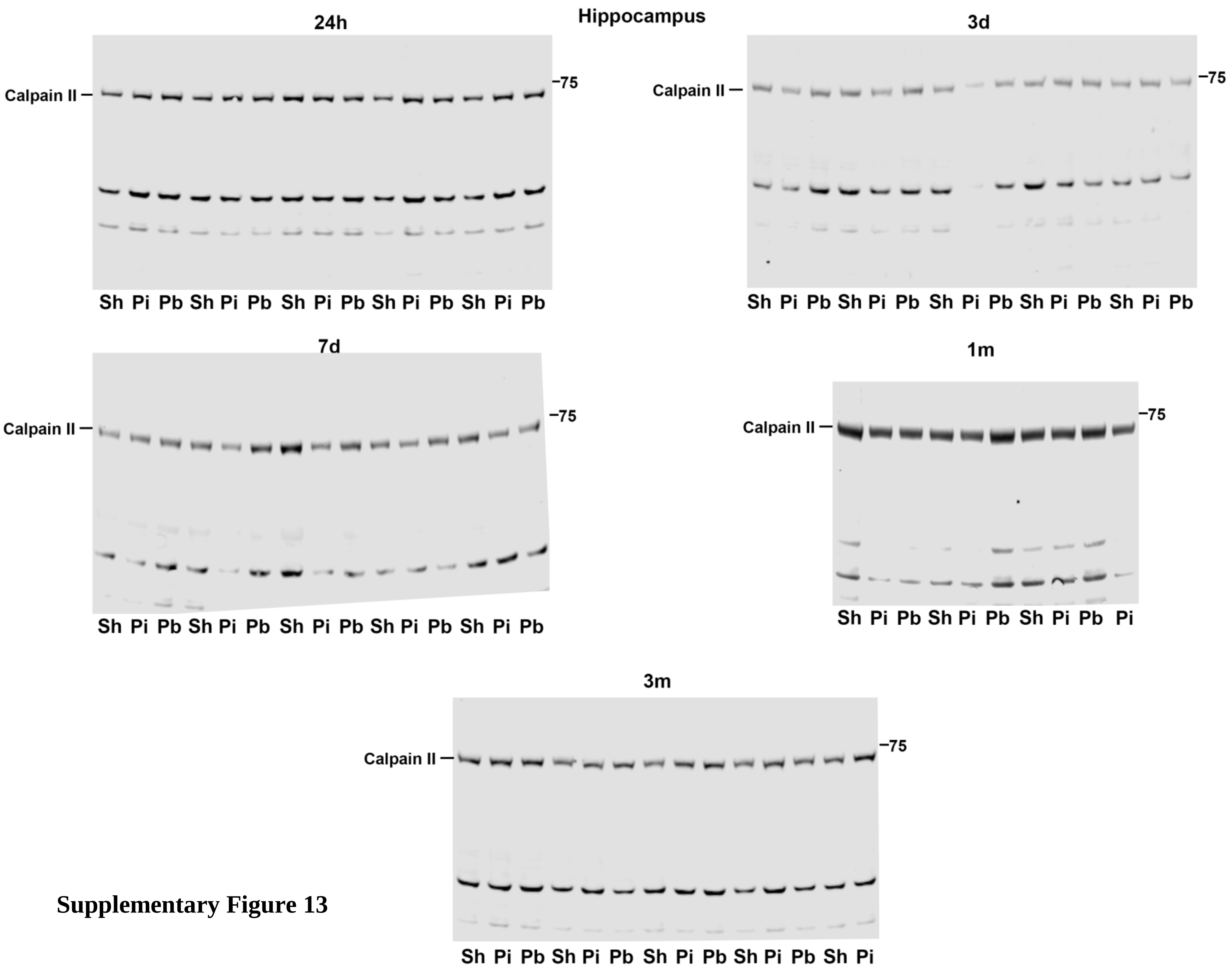

Supplementary Figure 13

Supplementary Figure 13: Full gel images of western blots for calpain-II in hippocampus from 24h – 3m, which were presented as cropped representative images in Figure 4. Lanes were loaded with Sham (Sh), Probe (Pi), or PBBI (Pb) samples as indicated.

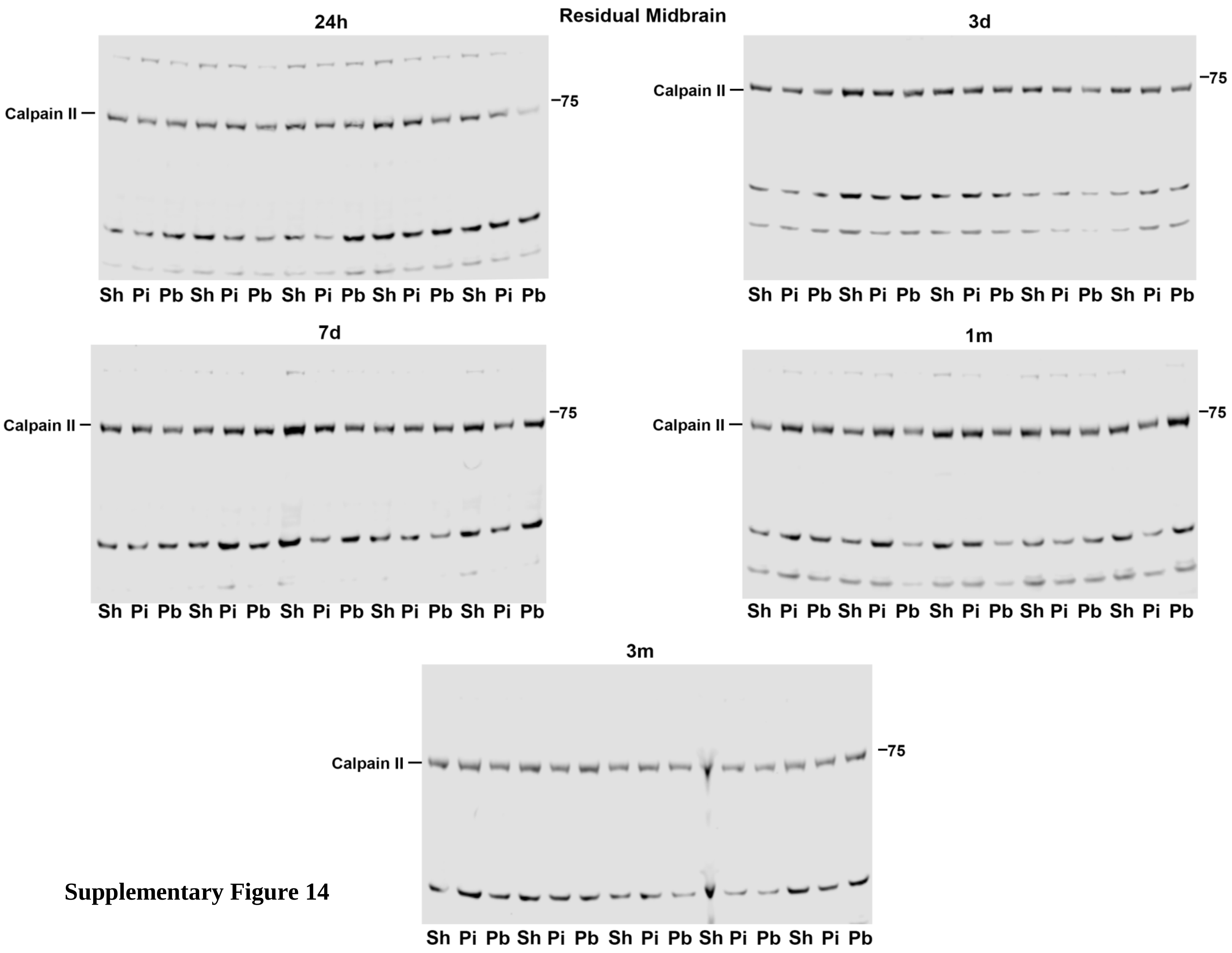

Supplementary Figure 14

Supplementary Figure 14: Full gel images of western blots for calpain-II in residual midbrain from 24h – 3m, which were presented as cropped representative images in Figure 4. Lanes were loaded with Sham (Sh), Probe (Pi), or PBBI (Pb) samples as indicated. The sample in lane 10 was rerun for quantitation purposes.

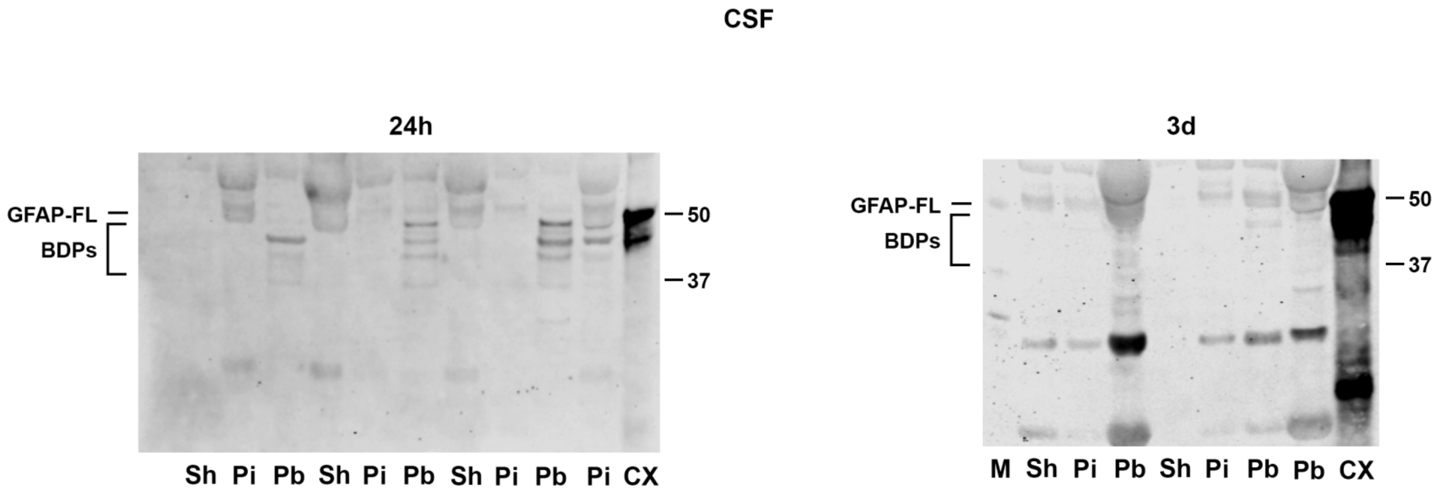

Supplementary Figure 15: Full gel images of western blots for GFAP and GFAP-BDPs in CSF from 24h and 3d, which were presented as cropped representative images in Figure 5. Lanes were loaded with Sham (Sh), Probe (Pi), or PBBI (Pb) samples as indicated. A cortex (CX) sample from a PBBI injured rat collected 24 hours after injury was also included as a positive control.

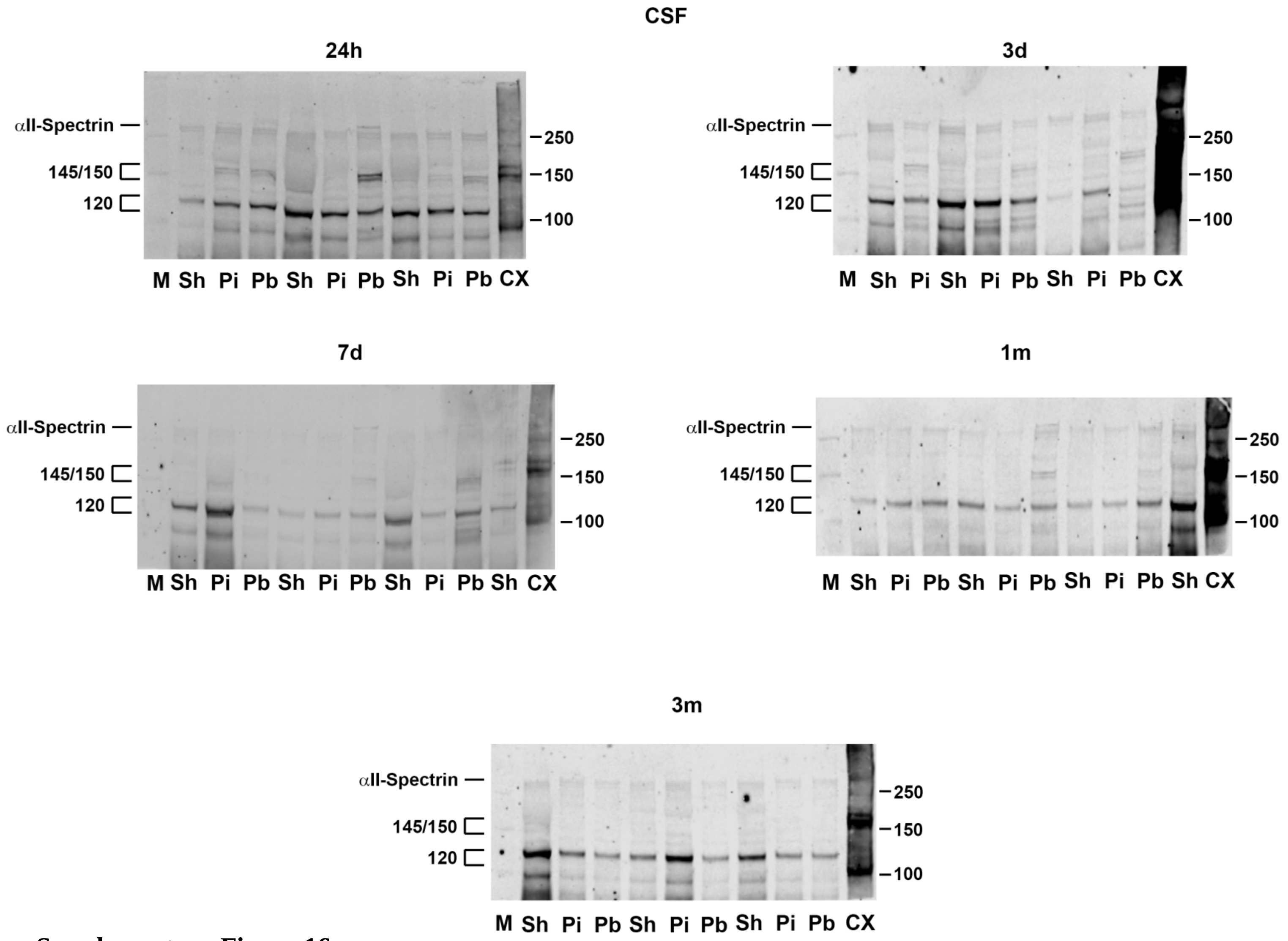

Supplementary Figure 16

Supplementary Figure 16: Full gel images of western blots for α-II spectrin and SBDPs in CSF from 24h – 3m, which were presented as cropped representative images in Figure 6. Lanes were loaded with Sham (Sh), Probe (Pi), or PBBI (Pb) samples as indicated. A cortex (CX) sample from a PBBI injured rat collected 24 hours after injury was also included as a positive control. The M denotes the molecular weight marker lane.

1.2 Supplementary Tables

Table S1: Correlation results between CSF and tissue levels of GFAP-FL and GFAP-BDPs by brain region and time point

	24h		3d	
	Pearson r	*p* value	Pearson r	*p* value
GFAP-FL (50 kDa)				
FCX	0.151	0.452	0.264	0.289
STM	0.267	0.179	0.436	0.070
HC	0.100	0.618	0.579	0.015*
RMB	0.030	0.882	0.263	0.293
GFAP-BDPs (37 - 48 kDa)				
FCX	0.569	0.002**	0.511	0.030*
STM	0.514	0.009**	0.678	0.002**
HC	-0.096	0.680	0.797	0.000***
RMB	0.476	0.016*	0.175	0.501

Supplementary Table 1: Pearson r correlation analyses were performed between levels of GFAP-FL and GFAP-BDPs in CSF and brain tissues by region and time point post injury. Protein levels were determined by Western blot at each time point post injury and were expressed as normalized values for CSF markers and as a percent sham for tissue markers for analysis. Sham, Probe, and PBBI injured rats were all included for analysis. Significant correlations are marked with *(p<0.05), **(p<0.01), or ***(p<0.001). CSF, cerebrospinal fluid; FCX, frontal cortex; STM, striatum; HC, hippocampus; RMB, residual midbrain.
